# Population dynamics of cross-protection against β-lactam antibiotics in droplet microreactors

**DOI:** 10.3389/fmicb.2023.1294790

**Published:** 2023-12-21

**Authors:** Xinne Zhao, Philip Ruelens, Andrew D. Farr, J. Arjan G. M. de Visser, Larysa Baraban

**Affiliations:** ^1^Institute of Radiopharmaceutical Cancer Research, Helmholtz-Zentrum Dresden-Rossendorf e. V. (HZDR), Dresden, Germany; ^2^Laboratory of Genetics, Wageningen University and Research, Wageningen, Netherlands; ^3^Department of Microbial Population Biology, Max Planck Institute for Evolutionary Biology, Plön, Germany

**Keywords:** droplet-based microreactors, bacterial cross-protection, β-lactam antibiotics, antibiotic resistance, cell filamentation

## Abstract

**Introduction:**

Bacterial strains that are resistant to antibiotics may protect not only themselves, but also sensitive bacteria nearby if resistance involves antibiotic degradation. Such cross-protection poses a challenge to effective antibiotic therapy by enhancing the long-term survival of bacterial infections, however, the current understanding is limited.

**Methods:**

In this study, we utilize an automated nanoliter droplet analyzer to study the interactions between *Escherichia coli* strains expressing a β-lactamase (resistant) and those not expressing it (sensitive) when exposed to the β-lactam antibiotic cefotaxime (CTX), with the aim to define criteria contributing to cross-protection.

**Results:**

We observed a cross-protection window of CTX concentrations for the sensitive strain, extending up to approximately 100 times its minimal inhibitory concentration (MIC). Through both microscopy and enzyme activity analyses, we demonstrate that bacterial filaments, triggered by antibiotic stress, contribute to cross-protection.

**Discussion:**

The antibiotic concentration window for cross-protection depends on the difference in β-lactamase activity between co-cultured strains: larger differences shift the ‘cross-protection window’ toward higher CTX concentrations. Our findings highlight the dependence of opportunities for cross-protection on the relative resistance levels of the strains involved and suggest a possible specific role for filamentation.

## 1 Introduction

**Figure 1:**
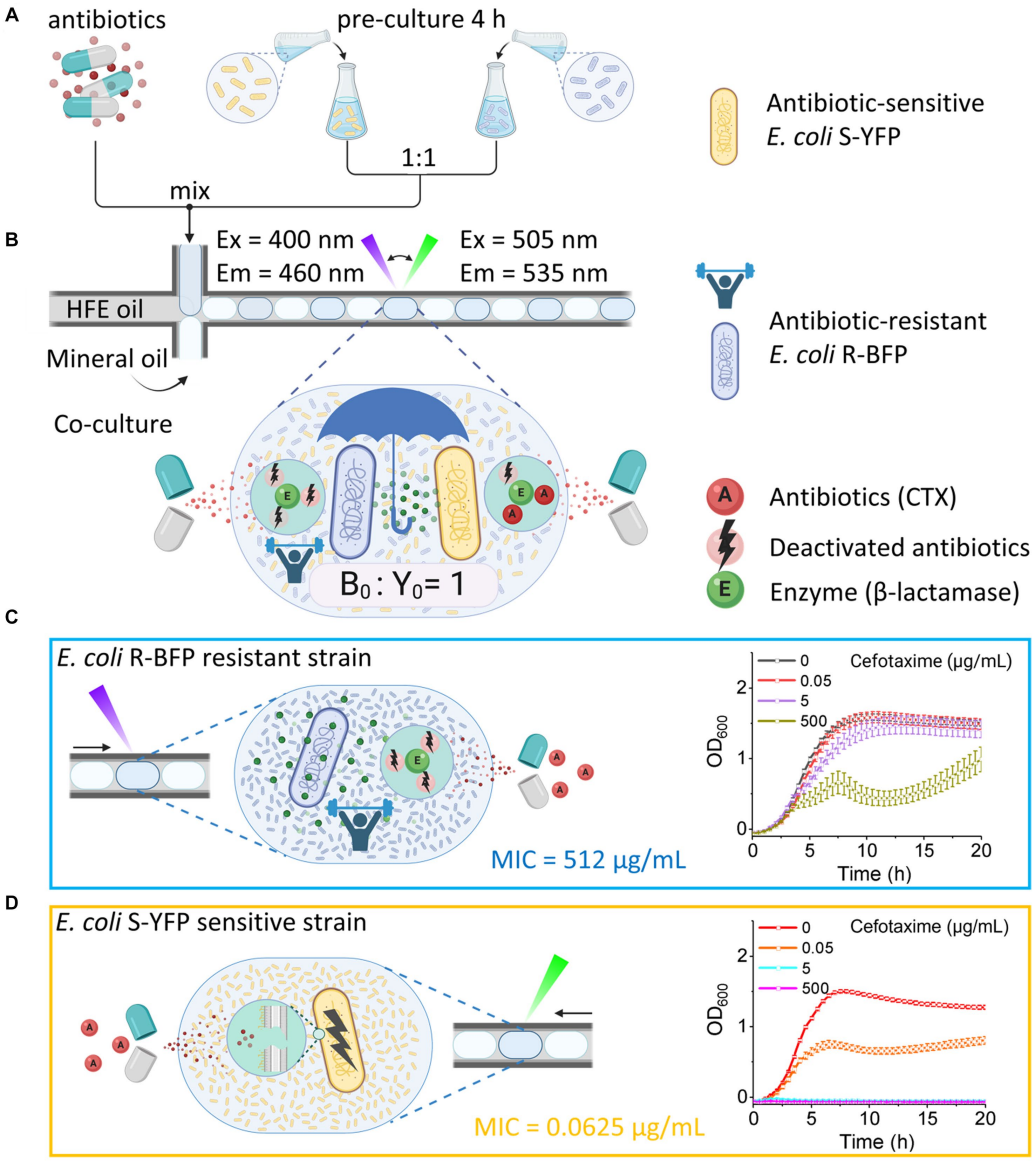
Quantifying bacteria growth in medium containing CTX in nanoliter droplet reactors. **(A)** Generating droplets in FEP tubing with HFE oil (continuous phase), mineral oil (blocker to prevent fusion of aqueous droplets), and aqueous phase droplets (antibiotic and bacteria in the M9 component medium). Bacteria were batch pre-cultured in flasks with M9 component medium for 4 h before being encapsulated in droplets. **(B)** Co-culture of antibiotic-resistant *E. coli* R-BFP and antibiotic-sensitive *E. coli* S-YFP in droplets with the initial inoculum ratio of 1:1 and a total cell number per drop of about 2,000. The growth of both bacterial strains was monitored by automatically switching the fluorescence detection modes (LEDs and optical filters) and the droplet moving direction. In the droplet, the β-lactamase produced by *E. coli* R-BFP reduces the CTX concentration, also helping *E. coli* S-YFP to survive. **(C)** Growth of a monoculture *E. coli* R-BFP was monitored by detecting the blue fluorescent protein with UV light. The β-lactamase produced by *E. coli* R-BFP inactivated CTX, helping it to survive. **(D)** Growth of monoculture *E. coli* S-YFP was monitored by detecting yellow fluorescent protein with cyan light. The β-lactam antibiotic CTX inhibited *E. coli* S-YFP growth. Created with BioRender.com.

Antibiotics are widely used in the clinic to treat bacterial infections (Aminov, 2010). However, the misuse and overuse of antibiotics in recent decades have contributed to a concerning rise in drug-resistant bacterial strains, resulting in a dramatic decline in antibiotic efficacy. Alarmingly, the mortality attributable to infections caused by antibiotic-resistant bacteria now exceeds the fatalities linked to HIV/AIDS or malaria (Bell, 2014; GBD 2019 Diseases and Injuries Collaborators, 2020; Antimicrobial Resistance Collaborators, 2022). Resistance not only ensures the survival of resistant strains within a bacterial community but the resistant strains can also facilitate the survival of sensitive strains at otherwise lethal antibiotic concentrations through cross-protection interactions (Figure 1). Cross-protection is the phenomenon in which the concentration of antibiotics in the environment is lowered due to the presence of antibiotic-resistant members of the population (Vega and Gore, 2014), and is often caused by active degradation of the antibiotic in question (Nicoloff and Andersson, 2016). A classic example of this phenomenon is the degradation of β-lactam antibiotics by co-existing β-lactamase expressing species (Maddocks and May, 1969). The study of cross-protection by β-lactamases is suitable because the process has been described both phenomenologically (Yurtsev et al., 2016) and theoretically (Geyrhofer et al., 2023). Although the implications of cross-protection on community dynamics are not yet fully understood (Balaban et al., 2004; Yu et al., 2022), recent research on multispecies coexistence in complex environments has shown that cross-protection can significantly shape the composition of bacterial communities in the presence of antibiotics (Pande et al., 2014; Medaney et al., 2016; Adamowicz et al., 2018). For instance, Yurtsev et al. (2016) demonstrated an effective mutualistic cross-protection interaction between two resistant *Escherichia coli* strains in a two-drug environment, allowing the co-culture to withstand high antibiotic concentrations at which neither of the two strains can survive alone. Furthermore, Frost et al. (2018) concluded that the density and spatial structure of bacterial colonies strongly influences the occurrence of cross-protection during co-culturing antibiotic-sensitive and -resistant strains. Enzymatic degradation of antibiotics is considered an important mechanism therein allowing resistant cells to protect sensitive cells (Nicoloff and Andersson, 2016; Kim et al., 2018; Geyrhofer et al., 2022). Notably, extensive research has been dedicated to the enzymatic degradation of β-lactam antibiotics due to their clinical importance and high efficiency against a broad spectrum of bacterial species (Angelis et al., 2020; Lima et al., 2020).

However, our current understanding of antibiotic cross-protection is limited in several respects. First, the specific conditions that lead to the emergence of cross-protection, such as the antibiotic dosage and the complexity of the environment, remain unclear. Second, the impact of bacterial morphology and metabolism has not been thoroughly explored. A deeper understanding of cross-protection will allow the development of more effective antibiotic therapies to combat bacterial infections.

Our study aims to deepen the understanding of the consequences of cross-protection on population dynamics by investigating the coexistence between two *E. coli* strains with substantial differences in β-lactamase activity within nanoliter droplets. To achieve this, we employ a high-throughput fluidic system (Zhao et al., 2021; Nguyen-Le et al., 2023) capable of efficiently monitoring the population dynamics of monocultures and mixed populations of bacterial strains in hundreds of droplets using integrated fluorescence detection modules. By comparing two strains with distinct resistance levels to the β-lactam CTX, we confirm the existence of a cross-protection window – a range of CTX concentrations surpassing the MIC of the sensitive strain that allows its survival. We subsequently assessed the effect of CTX degradation in droplets – caused by β-lactamase activity from resistant strains in co-culture - on the survival of sensitive strains (Bottery et al., 2021; Figure 1B). To better understand our findings, we employ a combination of techniques, including microscopy, fluorescence, and β-lactamase activity measurements. Our observations indicate that when bacteria are exposed to high levels of antibiotics, cells first swell, then elongate into filaments, and eventually lyse. These morphological alterations coincide with enhanced secretion of β-lactamase, either via cell lysis or increased cell permeability. Notably, the cross-protection window is dependent on both the antibiotic dosage and the difference in β-lactamase activity between co-cultured strains.

## 2 Materials and methods

### 2.1 Bacterial samples preparation

#### 2.1.1 Bacterial strains

Ancestral *E. coli* strains of this study - MG1655 galK::SYFP2-FRT-*cat*-FRT, and MG1655 galK::mTagBFP2-FRT-*cat*-FRT - were kindly sent by the Andersson lab and the construction of the fluorescent marker has been previously described (Gullberg et al., 2014). The chloramphenicol marker was removed by temporary expression of FLP recombinase resulting in MG1655 galK::SYFP2-FRT (termed S-YFP) and MG1655 galK::mTagBFP2-FRT (termed S-BFP). A clonal isolate of S-BFP was then modified at chromosomal *galK* to express TEM-1 β-lactamase genes, as previously described (Farr et al., 2023). This resulted in strains R-BFP@4 (MG1655 galK::mTagBFP2-FRT TEM1-G238S), *E. coli* R-BFP@8 (MG1655 galK::mTagBFP2-FRT TEM1-M182T-G238S), and *E. coli* R-BFP@512 (MG1655 galK::mTagBFP2 TEM1-E104K-M182T-G238S). These mutants of TEM-1 derived from a previous study (Schenk et al., 2013). TEM1 is a β-lactamase, which efficiently hydrolyzes penicillin-type β-lactam antibiotics (Munita and Arias, 2016; Ruiz, 2018) M182T, E104K, and G238S are mutations in TEM1 β-lactamase that - upon combination of these mutations - allow increased hydrolysis of cefotaxime, a third-generation cephalosporin (Palzkill, 2018).

#### 2.1.2 Bacteria activate culture and storage

1 mL 70% glycerol (v/v %) was added to the bacterial cells (collecting by centrifuging and removing the supernatant of bacterial culture media) was added to the bacterial cells (collected by centrifuging and removing the supernatant of bacterial culture media) in 1.5 mL Eppendorf tubes before freezing before freezing in a freezer at −80°C for long-term storage of bacterial stocks as bacterial stocks. Every week, one of the frozen stocks was unfrozen and transferred to a 100 mL flask with a 50 mL M9 compound medium and incubated overnight at 37°C with 170 rpm shaking speed for recovery. The activated bacterial medium was then stored in the fridge at 4°C for temporary usage (for a maximum of one week). Before each experiment, 200 μL of this activated bacterial medium was transferred to a 50 mL flask containing 25 mL M9 compound medium and incubated at 37°C with 170 rpm shaking speed for 4 h to reach the mid-exponential phase.

### 2.2 Medium and antibiotic preparation

#### 2.2.1 M9 compound medium preparation

In this work, the M9 medium was chosen for bacterial cultivation and detection to avoid fluorescent signal interference from the medium. For preparing the M9 salt solution, 200 mL 5× M9 minimal salt solution (sterile, Sigma Aldrich) was added to 800 mL Miller Q water (autoclaved at 121°C for 20 min) to get the 1× M9 minimal salt solution. For preparing the M9 compound medium, 8 g glucose (D-glucose, Sigma Aldrich), 1 g MgSO_4_‧7H_2_O (Sigma Aldrich), and 0.5 g casein hydrolysate (Sigma Aldrich) were dissolved in 50 mL 1× M9 minimal salts solution and filtered with a sterile syringe and filter (VWR, 0.2 μm cellulose acetate membrane). Then, a 50 mL filtered M9 solution that contains the nutrients was added to a 950 mL 1× M9 minimal salts solution.

#### 2.2.2 Antibiotic stock solution preparation

The solvent used to prepare and dilute antibiotic Cefotaxime sodium salt (CTX, Sigma Aldrich) was the M9 compound medium to avoid the concentration of the nutrients decreasing in the prepared culture medium. The original antibiotic stock solution (25 mg/mL CTX solution) was prepared by dissolving 66.49 mg Cefotaxime sodium salt (potency is 940 μg/mg) into 2.5 mL M9 compound medium (Andrews, 2001). Afterward, the original CTX solution was filtered (VWR, 0.2 μm cellulose acetate membrane) and further diluted to various gradient concentrations. In the following, 300 μL solution with different concentrations was transferred in 500 μL Eppendorf tubes with correlated concentration labels. All the prepared antibiotic stock solutions were kept in the freezer at −20°C for a maximum of 2 months. To enable the expression of all TEM motifs under the control of *lacI* repressor, 50 μM isopropyl β-D-1-thiogalactopyranoside (IPTG, Sigma Aldrich) was also added to the growth medium for each experiment (Gomes et al., 2020). The IPTG stock (5 mM) was prepared by dissolving 119.15 mg into a 100 mL M9 compound medium, followed by transferring 1 mL to 2 mL Eppendorf tubes and keeping it in the freezer at −20°C for a maximum of 2 months.

#### 2.2.3 Nitrocefin working solution stock preparation

Nitrocefin working solution stock (500 μg/mL) was first prepared by dissolving 5 mg nitrocefin in 500 μL dimethyl sulfoxide (DMSO, Sigma Aldrich) and followed by adding 9.5 mL phosphate buffer (100 mM, pH 7) to obtain a total volume of 10 mL. 500 μL of the stock solution was transferred into each 1 mL brown Eppendorf tube and kept in a freezer at −20°C for a maximum of 2 months.

### 2.3 Minimum inhibitory concentration determination

For each strain (for *E. coli* S-YFP and *E. coli* R-BFP variants), the MIC of CTX was detected in duplicate. 200 μL of fresh M9 composition medium containing 50 μM IPTG was added to each well of a clear 96-well microtiter plate and inoculated with activated bacteria 0.01 A (5.0 × 10^6^ cells/mL). The CTX concentration gradient was prepared by diluting 1,024 μg/mL in 18 successive 2-fold dilutions, ranging from 1,024 μg/mL to 0.0039 μg/mL. The microplates were incubated at 37°C for 24 h and then subjected to optical density testing, with the MIC determined at OD600 > 0.2 A (1.0 × 10^8^ cells/mL). The MIC values (Table 1) obtained in this work were higher than in other research (Ruelens and De Visser, 2021) due to the addition of IPTG and higher inoculum (Saebelfeld et al., 2022).

**Table 1:** Strains and genotypes.

Strain	Genotype	MIC (μg/mL)	Ref.
*E. coli* S-YFP	*E. coli* MG1655 *galK*::SYFP2-FRT	0.0625	Gullberg et al. (2014)
*E. coli* R-BFP4	*E. coli* MG1655 *galK*::mTagBFP2-FRT-*TEM-1* G238S	4	Farr et al. (2023)
*E. coli* R-BFP8	*E. coli* MG1655 *galK*::mTagBFP2-FRT- *TEM-1* E104K G238S	8	Farr et al. (2023)
*E. coli* R-BFP512	*E. coli* MG1655 *galK*::mTagBFP2-FRT-*TEM-1* E104K M182T G238S	512	Farr et al. (2023)

### 2.4 Bacterial growth monitor in nanoliter droplet reactors

To prepare the initial bacterial samples of nanoliter droplet reactors, bacteria were firstly pre-cultured for 4 h to reach the mid-exponential growth phase (37°C, 170 rpm). Then, the bacteria were diluted to 1.0 × 10^7^ cells/mL (0.0196 A, OD_600_, Eppendorf Biophotometer) with the fresh M9 compound medium (stored at 4°C) in a 1.5 mL Eppendorf tube. Next, the diluted bacterial medium was refilled into a 5 mL syringe and fixed on the Nemesys pump (CETONI GmbH). For monoculture, the single strain (*E. coli* R-BFP or *E. coli* S-YFP) of the bacterial medium (1.0 × 10^7^ cells/mL, 0.0196 A) was mixed with M9 component media (containing 100 μM IPTG and two times the designed concentration of CTX) by pumping them into the Fluorinated ethylene-propylene (FEP) tubing with the same flow rate to form droplets (200 nL, with an initial cell density of 5.0 × 10^6^ cells/mL) (Baraban et al., 2011). For co-culture, before encapsulation, the strains (both *E. coli* R-BFP and *E. coli* S-YFP) were separately pre-culture for 4 h in M9 medium and subsequently diluted and mixed with a ratio of 1:1 (both cell densities are 1.0 × 10^7^ cells/mL). The mixed bacterial medium was injected into the tubing with the same volume of M9 component media (containing 100 μM IPTG and two times the designed concentration of CTX, from 0 to 1,000 μg/mL). The initial cell density of both bacterial strains in the droplets was 5.0 × 10^6^ cells/mL. To avoid clumping bacteria, autoclaved magnetic stirring bars (diameter = 2 mm and length = 5 mm) were placed into the syringes (5 mL, diameter = 10.301 mm, length = 60 mm) near a magnetic stirrer with the speed of 500 rpm during the droplet generation. For each experiment, a droplet sequence with approximately 450 droplets was generated and incubated in the system at 37°C for 20 h. UV LED (385 nm, Thorlabs) with optical filters (*Em*/*Ex* = 400 nm/460 nm) was used to detect *E. coli* R-BFP while pushing through the detection area. When the droplet sequence went backward (refill mode) through the optical detectors, cyan LED (490 nm, Thorlabs) with optical filters (*Em*/*Ex* = 505 nm/535 nm) was chosen to detect *E. coli* S-YFP.

### 2.5 Bacterial growth monitoring in plate reader

#### 2.5.1 Samples preparation

Bacterial samples grown in the microplate were prepared by pre-culture activated bacteria for 4 h and first diluted to 5.0 × 10^7^ cells/mL with M9 compound medium. In microplates, each well was filled with 20 μL bacterial medium (monoculture: 20 μL *E. coli* S-YFP or *E. coli* R-BFP; co-culture: 20 μL *E. coli* S-YFP and 20 μL *E. coli* R-BFP), 20 μL IPTG (500 μM), 20 μL CTX (10 times the final concentration), and fresh M9 compound medium to a total volume of 200 μL. Then the microplates (with a final inoculate of 5.0 × 10^6^ cells/mL) in the plate reader (Cytation 5, Biotek) were incubated at 37°C for 20 h and measured every 5 min (samples were shaken before each measurement).

#### 2.5.2 Cell density measurement

Cell density was measured by recording the absorption at 600 nm wavelength in transparent 96 well plates.

#### 2.5.3 Fluorescence intensity measurement

Fluorescence intensity was measured in a black 96-well plate. For measuring the R-BFP, the excitation wavelength was 400 nm ± 20 nm, and the emission wavelength was 460 nm ± 20 nm. For measuring S-YFP, the excitation wavelength was 490 nm ± 20 nm, and the emission wavelength was 530 nm ± 20 nm. After setting the excitation and emission wavelength for measuring R-BFP and S-YFP, the fluorescence intensity-bacterial concentration calibration curves were first obtained by detecting the bacterial fluorescence signal with different cell concentrations. To ensure the fluorescence signal truly reflects the viable cell concentrations, the bacteria were first cultured in the fresh M9 component medium for 8 h before diluting to various concentrations with gradients.

### 2.6 Cell shape and size observation by fluorescence microscopy

#### 2.6.1 Samples preparation

To prepare samples for microscope observation, activated bacteria of *E. coli* S-YFP (sensitive strain) and *E. coli* R-BFP (resistant strains) were firstly pre-cultured for 4 h. Then the bacterial pre-grown medium was diluted to a final inoculate of 5.0 × 10^6^ cells/mL with an M9 component medium containing 50 μM IPTG and different concentrations of CTX (e.g., 0, 0.05, 0.5, 5, 50, and 500 μg/mL). After incubating samples in Eppendorf tubes (2 mL) for 20 h at 37°C, the bacterial medium was diluted with PBS solution based on the needs and dropped on glass slides. For each experiment, a 5 μL sample was dropped on a clean glass slide (22 mm × 40 mm) by a 0.1–10 μL pipette; later, a cover glass slide (22 mm × 22 mm) was covered on top of the sample. After a few minutes, until all the bacteria stopped flowing between two glass slides, images of the bacteria were taken under bright field, BFP mode, and GFP mode with different magnifications.

#### 2.6.2 Fluorescence microscope optical set for bacteria observation

For fluorescence microscopy (Axiovert 200 M, Carl Zeiss), the R-BFP mode with excitation wavelength 380 nm and emission wavelength 439 nm was utilized to observe *E. coli* R-BFP. Filter Set GFP with excitation wavelength 489 nm and emission wavelength 509 nm was used to observe *E. coli* S-YFP.

### 2.7 Cell-free medium fluorescence intensity detection

The pre-grown bacteria (monoculture with an inoculum of 5.0 × 10^6^ cells/mL, co-culture with an inoculating ratio of 1:1) was cultured in an M9 component medium with 50 μM IPTG and different concentrations of CTX for 20 h. Then the bacterial medium was separated into cell-free medium and pure cells by following steps, (1) centrifuging (3,370 rpm, 5 min) growth culture media to settle down cells and get supernatants; (2) filter sterilizing (0.2 μm syringe filter) the supernatants to remove the residual cells; (3) collect the filtrate. The pelleted cells were then re-suspended in the fresh M9 component medium. Afterward, the original bacterial medium, cell-free medium, and re-suspended pure cells were pipetted to a 96-well plate (200 μL per well) and measured the fluorescence intensity by a plate reader (R-BFP: *Ex* = 400 ± 20 nm, *Em* = 460 ± 20 nm; S-YFP: *Ex* = 490 ± 20 nm, *Em* = 530 ± 20 nm).

### 2.8 β-Lactamase activity testing

The β-lactamase activity testing was done by nitrocefin assay in the cell-free medium. Bacterial samples were prepared using the same protocol as the microscopic observation sample preparation. The β-lactamase activity was quantified by the red absorption (OD_490_) change per minute (Diene et al., 2019). The slope of OD_490_-Time was used for describing the β-lactamase activity among the samples.

#### 2.8.1 β-Lactamase activity (released to extra-cell) of cell-free spent media measurement

Before incubating and after incubating for 20 h, the bacterial medium was collected and centrifuged (3,370 rpm, 5 min). Then the supernatants were taken and filtered through a sterile filter (0.2 μm syringe filter) to get a cell-free spent medium. Afterward, the medium was kept on ice throughout the experiment. In a 96 well-plate, 100 μL of 10 μg/mL nitrocefin (diluting 500 μg/mL working solution stock by fresh M9 compound media) was added to 100 μL cell-free medium in each well. Red absorption (OD_490_) was measured in a plate reader every minute for 1 h as the nitrocefin was hydrolyzed. Besides, the samples with a high absorption signal were diluted to ensure the results were in the nitrocefin assay range.

#### 2.8.2 Cell wall break treatment of entire cell media for β-lactamase activity measurement (production)

The cell wall break treatment was done by the frozen treatment. After incubating for 20 h, the entire bacterial cell medium was collected. One half was centrifuged and filtered to get a cell-free spent medium as a control group, the rest half was stored in a freezer at −20°C for 2 h; then unfroze at room temperature for 30 min and repeated twice. Then 100 μL of the entire cell media after cell wall break treatment was added into a 96 well-plate followed by adding 100 μL of 10 μg/mL nitrocefin and measuring red absorption (OD_490_).

#### 2.8.3 β-lactamase activity per unit cell biomass calculation

Considering that cell density (Supplementary Figure S1A) and cell length (Supplementary Figures S2A–C) changed due to different antibiotic stress, β-lactamase activity per unit cell biomass (Supplementary Figure 2D, mean cell volume × cell density) is used to compare the fraction between β-lactamase activity release (cell-free spent media) and β-lactamase activity production (entire cell media after frozen cell wall break treatment), as shown in Supplementary Figure 2E.

### 2.9 Cell distance estimation

Since the bacteria are uniformly distributed in the droplet environment, and the initial inoculum of 2000 cells is considered to occupy the droplet equally, then the initial volume occupied by each cell can be calculated as follows:


(S1) $$V_{1}=\;\frac{V_{\mathrm{droplet}}}{\mathrm{initial}\;\mathrm{cell}\;\mathrm{number}}=\frac{4}{3}\pi r_{1}^{3}$$

Where *V_1_* is the average initial cell move space, *V_droplet_* is 200 nL, the *initial cell number* is 2000 cells, and *r_1_* is the average initial cell move space radius.

According to Eq. S1, the *r_1_* is calculated to be 28 μm. Thus, the average estimated cell distance is around 56 μm.


(S2) $$V_{2}=\;\frac{V_{\mathrm{droplet}}}{\mathrm{final}\;\mathrm{cell}\;\mathrm{number}}=\frac{4}{3}\pi r_{2}^{3}$$

Where *V_2_* is the average final cell move space, *V_droplet_* is 200 nL, the *final cell number* is assumed as 2 × 10^7^ cells, and *r_2_* is the average final cell move space radius.

According to Eq. S2, the *r_2_* is calculated to be 1.34 μm. Thus, the average estimated cell distance is around 2.68 μm.

## 3 Results and discussion

### 3.1 Investigating bacterial growth and competition using nanoliter droplet reactors

To investigate the effect of antibiotics on community dynamics, we tracked the growth of an antibiotic-sensitive (minimum inhibitory concentration MIC_CTX_ = 0.0625 μg/mL, see bacteria and genotypes in Table 1) and antibiotic-resistant (MIC_CTX_ = 512 μg/mL) strain in co-culture in *ca.* 500 droplet reactors (volume of each droplet is 200 nL) across various doses of CTX. The antibiotic-sensitive strain, devoid of β-lactamase activity, was modified to express an SYFP2 gene, producing a yellow fluorescent protein that allows visualization of cells and quantification of cellular growth. Similarly, the antibiotic-resistant strain expresses mTagBFP2, a blue fluorescent protein, and a β-lactamase that efficiently hydrolyzes cefotaxime β-lactam antibiotics (see Methods for details). The experiment involved encapsulating the two *E. coli* strains (yellow and blue fluorescence bacteria) into aqueous droplets with an inoculum ratio of 1:1, and a total initial cell number of approximately 2,000. Aqueous droplets, containing nutrients, bacteria and antibiotics, were generated through co-infusing the antibiotic-bacteria solution, mineral oil (as a spacer), and HFE oil with 1% surfactant PicoSurf 2TM (continuous phase) into a cross-junction at a flow rate ratio of 5:5:1 (mL/h).

**Figure 2:**
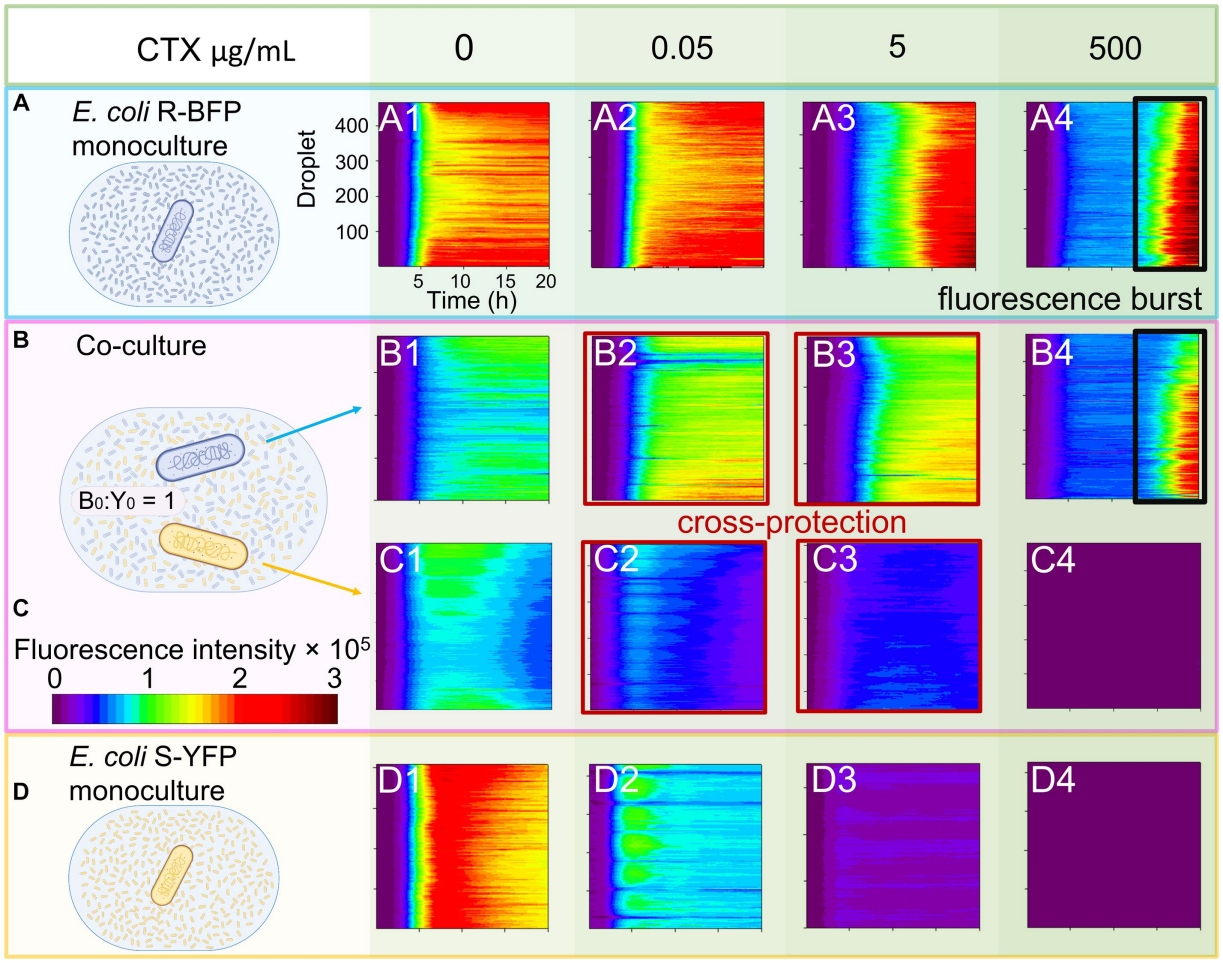
Monoculture and co-culture population dynamics of *E. coli* S-YFP and *E. coli* R-BFP in nanoliter droplet reactors with varying CTX concentrations. Fluorescence heat maps show the growth and death of both strains during for 20 h in approximately 400 droplets. **(A)** Monocultures of *E. coli* R-BFP incubated with an initial cell density of 1,000 cells/droplet with different CTX concentrations of 0 **(A1)**, 0.05 **(A2)**, 5 **(A3)**, and 500 **(A4)** μg/mL. Co-cultures of **(B)** *E. coli* R-BFP and **(C)** *E. coli* S-YFP incubated with initial cell density ratios of 1,000:1000 cells/droplet: cells/droplet with different CTX concentrations of 0 (**B1** and **C1**), 0.05 (**B2** and **C2**), 5 (**B3** and **C3**), and 500 (**B4** and **C4**) μg/mL. **(D)** Monocultures of *E. coli* S-YFP incubated with initial cell densities of 1,000 cells/droplet with different CTX concentrations of 0 (**D1**), 0.05 (**D2**), 5 (**D3**), and 500 (**D4**) μg/mL. The time scale is from 0 to 20 h. The fluorescence intensity scale is from 0.0–3.0 × 10^5^ a.u.

A millifluidic reactor system (Zhao et al., 2021) with two integrated fluorescence channels was utilized to monitor the growth of each bacterial strain in individual droplets in real-time by parallel detection of their fluorescent emissions (Figure 2). Initially, the properties and response to antibiotics of *E. coli* R-BFP (resistant BFP, Figure 1C) and *E. coli* S-YFP (sensitive YFP, Figure 1D) were examined in monoculture. It was observed that *E. coli* S-YFP exhibited growth inhibition at CTX concentrations above 0.05 μg/mL, while *E. coli* R-BFP survived even at CTX concentrations of up to 500 μg/mL. The growth kinetics of monoculture *E. coli* R-BFP are summarized in color maps in Figure 2A, A1–A4. In the absence of the antibiotic, the fluorescence intensity rapidly increased after a short lag phase until the stationary phase was reached. As the CTX concentration increased from 0.05 μg/mL to 5 μg/mL, the impact of antibiotics became increasingly apparent, primarily manifesting as increased lag phase duration (Supplementary Figure S3). Further increase of CTX concentration to 50 μg/mL (Supplementary Figures S3F–H) and 500 μg/mL (close to its MIC at 512 μg/mL) resulted in the stationary phase fluorescence stabilizing below 1.0 × 10^5^ a.u. (between 5 and 15 h of incubation); Notably, after 15 h of incubation, the fluorescence intensity in droplets significantly increased, leading to the occurrence of so-called signal bursts (Figure 2, A4). Conversely, the growth of the sensitive strain was strongly affected by CTX at 0.05 μg/mL (near its MIC at 0.0625 μg/mL, Table 1), as illustrated in Figure 2D, D1–D4. Remarkably, even at CTX concentrations of 5 to 10 times the MIC of S-YFP (Supplementary Figures S4C,D), bacteria still showed minimal growth. However, upon further increasing the CTX to 5 μg/mL and higher, microbial growth of the sensitive strain was completely suppressed.

Once both strains were mixed in the droplet reactor, as described above and in the Methods section, they distributed uniformly in the culture medium. We estimated the initial distance between bacterial cells to be on average 50–60 μm (5.0 × 10^6^ cells/mL, see Methods: Cell distance estimation) which decreases as bacterial growth progresses (down to 2.68 μm without antibiotic stress). The distance between bacteria might be important as the continuous release of β-lactamase could potentially play a critical role in the emergent phenomena of cross-protection among bacteria in close proximity (Frost et al., 2018). If this holds true, increases in S-YFP fluorescence should be detectable in the droplets with the growth kinetics of both strains in droplet co-cultures exhibiting behavior influenced by both (a) competition for nutrients and space and (b) inhibition by CTX (Figures 2B,C).

### 3.2 Emergence of cross-protection in bacterial co-cultures

In the absence of antibiotics (no inhibition, Figure 2, B1, C1), competition between the strains dominates, leading to a decrease in cell concentration (reflected as fluorescence intensity) to half the level observed in monoculture conditions (compare Figure 2, A1, D1). The fluorescence bulk signal of cells in the stationary phase remained nearly equal, indicating that both strains have equal resource competitive ability in the absence of CTX. With the addition of low concentrations of CTX (0.05–5 μg/mL), the balance between S-YFP and R-BFP in the stationary phase caused a slight increase in fluorescence intensity for *E. coli* R-BFP due to the inhibition of S-YFP in co-culture conditions (Figure 2, B2, B3). However, this situation changes once the antibiotic concentration exceeds 50 μg/mL. Comparison of the fluorescence-time heat maps of *E. coli* R-BFP monoculture and co-cultures in Figure 2, A4, B4 (Supplementary Figure S5) reveals similar levels of fluorescence and ‘signal bursts’ after 15 h of incubation (reflecting a growth curve with late growth due to the breakdown of CTX, discussed below). At high CTX concentrations, competition between both strains was considerably weaker due to the antibiotic induced death of S-YFP.

The growth of *E. coli* S-YFP was influenced by the R-BFP strain and the presence of antibiotics (Figure 2, C2–C4 and Figure 2, D2–D4). In co-culture conditions with low CTX concentrations of 0.05 μg/mL (Figure 2, C2), the fluorescence signal of *E. coli* S-YFP appeared to be lower than in monoculture (panel D2), which is due to competition between S-YFP and R-BFP. However, if we regard 50% of the monoculture population size as a criterion (as R-BFP will also grow to 50%), the presence of R-BFP allows a larger population size of S-YFP than in its absence (S-YFP signals in coculture >50% of their monoculture, panel C2 and D2). Moreover, we observed visible growth of S-YFP in co-culture at a CTX concentration of 5 μg/mL (previously not observed in monoculture). This indicates that the antibiotic-sensitive strain could grow 100-fold its MIC due to the apparent protective influence of R-BFP (Figure 2, C3).

No further increase in fluorescent signal of strain S-YFP was observed at CTX doses above 50 μg/mL (Figure 2, C4; Supplementary Figure S6, for 50 μg/mL), suggesting no observable cross-protection provided by *E. coli* R-BFP at these higher CTX concentrations. To define the conditions for cross-protection more precisely, we introduce the concept of the cross-protection window - a range of antibiotic concentrations within which cross-protection is observed from increases in the fluorescent signal of the planktonic cells of sensitive strain. In our experimental setup with two microbial strains with distinct β-lactamase activities, the cross-protection window for the sensitive strain (MIC = 0.0625 μg/mL) was experimentally observed to span between 0.05–5 μg/mL.

To validate our findings, we reproduced these results using a 96-well microplate setup (Supplementary Figure S7). ‘Signal bursts’ were observed during late stationary phase when culturing the microbes with CTX concentrations close to their MICs (Figure 2, A4, B4), mirroring the results observed in the droplet reactors. Additionally, the OD_600_ measurement of monoculture *E. coli* R-BFP exhibited distinct changes in the growth kinetics at specific CTX concentrations (Supplementary Figure S8). When culturing in 50, 250, and 500 μg/mL CTX, biomass initially increased, followed by a decline and subsequent dramatic increase after 15 h of incubation. In contrast, *E. coli* S-YFP did not exhibit any specific patterns across all tested antibiotic concentrations. Considering the unexpected bi-phasic fluorescence curve observed in both droplets and plates, we conclude that at certain antibiotic concentrations and late incubation stages, the fluorescence intensity is no longer proportional to the biomass as determined by the optical density of the culture. This is further supported by Supplementary Figure S9 and Yoon et al. (2015) work.

### 3.3 Filamentation as a key factor in cross-protection

**Figure 3:**
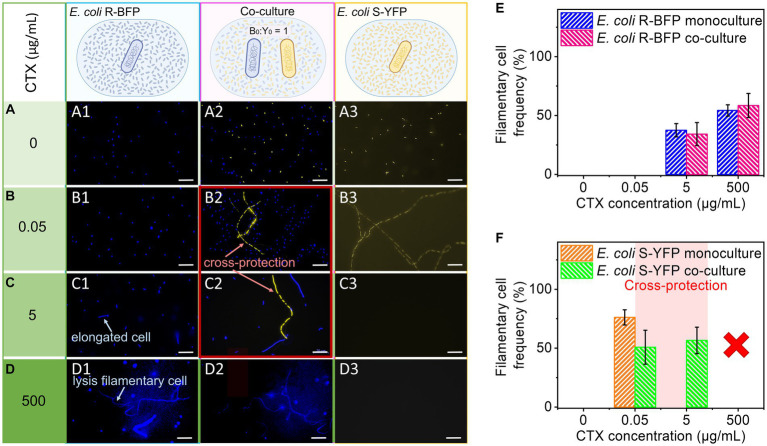
Microscopic observation and statistics of filamentation. *E. coli* R-BFP and *E. coli* S-YFP monoculture and co-culture in different concentrations of CTX **(A)** 0 µg/mL, **(B)** 0.05 µg/mL, **(C)** 5 µg/mL, and **(D)** 500 µg/mL were observed with fluorescence microscopy (20 h incubation) with 100× magnification. *E. coli* R-BFP monoculture in CTX with concentrations of 0 (**A1**), 0.005 (**B1**), 5 (**C1**), and 500 (**D1**) μg/mL (from top to bottom shown in different green colors from light to dark). *E. coli* R-BFP and *E. coli* S-YFP co-culture in CTX with concentrations of 0 (**A2**), 0.005 (**B2**), 5 (**C2**), and 500 (**D2**) μg/mL. *E. coli* S-YFP monoculture in CTX with concentrations of 0 (**A3**), 0.005 (**B3**), 5 (**C3**), and 500 (**D3**) μg/mL. Cell number and statistical morphology analysis: counting cell number and measuring cell length under a microscope after incubation for 20 h. Cells with a cell length greater than 4 μm are considered filamentary cells. **(E)** *E. coli* R-BFP monoculture and co-culture (with *E. coli* S-YFP with initial cell biomass ratio 1:1) filamentary cell frequency (filamentary cells as a percentage of total cells). **(F)** *E. coli* S-YFP monoculture and co-culture (with *E. coli* R-BFP with initial cell biomass ratio 1:1) filamentary cell frequency filamentary cells as a percentage of total cells. The total cell number (*n*) used to analyze cell morphology in monoculture and co-culture for each CTX condition is shown in Supplementary Table S1. Error bars are the standard deviation from 3 replicates.

We next conducted fluorescence microscopy to examine cell morphology and gain insights into the mechanism underlying the discrepancy between cell number and fluorescence as mentioned in the previous paragraph. Monocultures and co-cultures of *E. coli* R-BFP and S-YFP at various CTX concentrations were observed following 20 h of incubation (Figure 3). This analysis revealed that culturing the resistant R-BFP strain in the presence of CTX led to cell elongation and swelling, known as filamentation (Filipiak et al., 2020), starting from 5 μg/mL of CTX (Figure 3, C1). Filamentous cells were defined as greater than twice the average cell length of *E. coli* (Zhao et al., 2021) in the absence of stress (taking into account cells about to divide). As the CTX concentration exceeded 25 μg/mL, filamentation of the cells became more evident (Supplementary Figure S10C). Additionally, at CTX concentration above 250 μg/mL (Supplementary Figure S10E), the fluorescence of some filamentary cells vanished compared to cells growing in an antibiotic-free environment (Figure 3, D1; Supplementary Figure S11A). In contrast, *E. coli* S-YFP cells exhibited significant filamentation even at CTX concentrations of 0.005 μg/mL, below the MIC level (Supplementary Figure S12; Figure 3, B3). The corresponding background fluorescence levels tended to increase, suggesting significant cell lysis and release of fluorescent protein (Figure 3, B3), which also explains the relatively high fluorescence in Figure 2, D2. Once the CTX concentration reached 0.5 μg/mL (Supplementary Figure S12F), filamentary cells underwent lysis, followed by the complete disappearance of fluorescent cells at higher concentrations (Figure 3, C3, D3).

Upon co-culturing both strains in the CTX concentration range of 0 to 500 μg/mL, we observed morphological changes in the sensitive S-YFP strain, specifically their filamentation behavior (Figure 3, B2, C2, and D2). In the absence of antibiotics (0 μg/mL), no significant cell morphological differences were observed between monoculture and co-culture. However, due to competition, the cell density of S-YFP decreased by half in the co-culture. At low cefotaxime concentration (0.05 μg/mL), shorter filaments formed compared to the monoculture, and the frequency of cell filamentation decreased in the co-culture. Moreover, compared to the monoculture, the presence of fluorescent filaments of S-YFP cells was observed in co-cultures at 5 μg/mL CTX, confirming their survival at concentrations 100-fold higher than their MIC. In contrast, the morphology of the R-BFP bacteria in co-culture was similar to that in monoculture, with a slight increase in cell elongation starting from 5 μg/mL CTX. Microscopic analysis of *E. coli* S-YFP cells co-cultured with *E. coli* R-BFP in the range of 0.05 to 5 μg/mL CTX confirmed the observed cross-protection, wherein cell filamentation could potentially play a role.

To understand the role of filaments in cross-protection, we analyzed the microscopy images and calculated the cell density and filament frequency (Figures 3E,F; Supplementary Figures S1A,C). At 0.05 μg/mL CTX, the t-test result shows a marginally non-significant difference (at *p* < 0.05) in the filamentary frequency of co-culture (M = 0.76133, SD = 0.0647) and monoculture (M = 0.508, SD = 0.1445), t (n-1 = 2) = 2.7714, *p* = 0.050259. It suggests, that at a low CTX concentration, except for cross-protection, competition may still take an important part between two strains. Pink zones in Figure 3F (Supplementary Figures S1C,D) indicate the CTX range cross-protection window (S-YFP signals in coculture are >50% of their monoculture signal already at [CTX] = 0.05 μg/mL). Considering the competition with *E. coli* R-BFP, the cell density of *E. coli* YFP in the co-culture case should decrease to about half compared to monoculture, however, it increased, indicating that in 0.05 μg/mL CTX concentration, cross-protection appears. Analyzing the co-cultured cell density (cells per area) revealed similar results as in fluorescence-time heat maps that in the absence of antibiotic, competition led to the cell density decreasing to nearly half of that in the monoculture [Supplementary Figure S1A (R-BFP) and S1C (S-YFP)]. When the CTX concentration was varied from 0 to 0.05 μg/mL, the cell density of *E. coli* S-YFP sharply dropped in both monoculture and co-culture conditions. However, in co-culture, *E. coli* S-YFP managed to maintain a higher number of viable cells at 0.05 and 5 μg/mL compared to 50% of monoculture (see inset in Supplementary Figure S1C). At the same time, *E. coli* R-BFP exhibited a slight increase in cell densities at low concentrations of CTX (from 0 to 0.05 μg/mL), indicating the dominance of these resistant cells in competition with S-YFP (Supplementary Figure S1A). As CTX concentrations increased further, there was a visible decline in cell density for both strains (Supplementary Figures S1A,C). Panels E and F in Figure 3 show the frequency of filaments of both strains as inferred from microscopic images. The filamentation frequency confirms that filamentation is closely linked to the stress level arising from β-lactams exposure (Justice et al., 2008; Windels et al., 2019). Consequently, the decreased frequency of filaments of *E. coli* S-YFP at 0.05 μg/mL in co-cultures (green bars in Figure 3F) suggests a reduction in antibiotic stress due to the presence of R-BFP.

Although filaments might confer increased tolerance to β-lactams (Zahir et al., 2020), it’s worth noting that filamentous cells ultimately lyse in the presence of the antibiotic. This can be observed in Figure 3, D1 (Supplementary Figures S11, S13; Supplementary Table S2), where an increase in antibiotic concentration decreased the fluorescence in some of the filamentous cells (Supplementary Figures S1B,D). We hypothesize that a high percentage of these non-fluorescent filaments indicate a greater likelihood of filamentous cells undergoing lysis and releasing fluorescent protein into the media.

### 3.4 Role of the enzyme leakage in cross-protection

**Figure 4:**
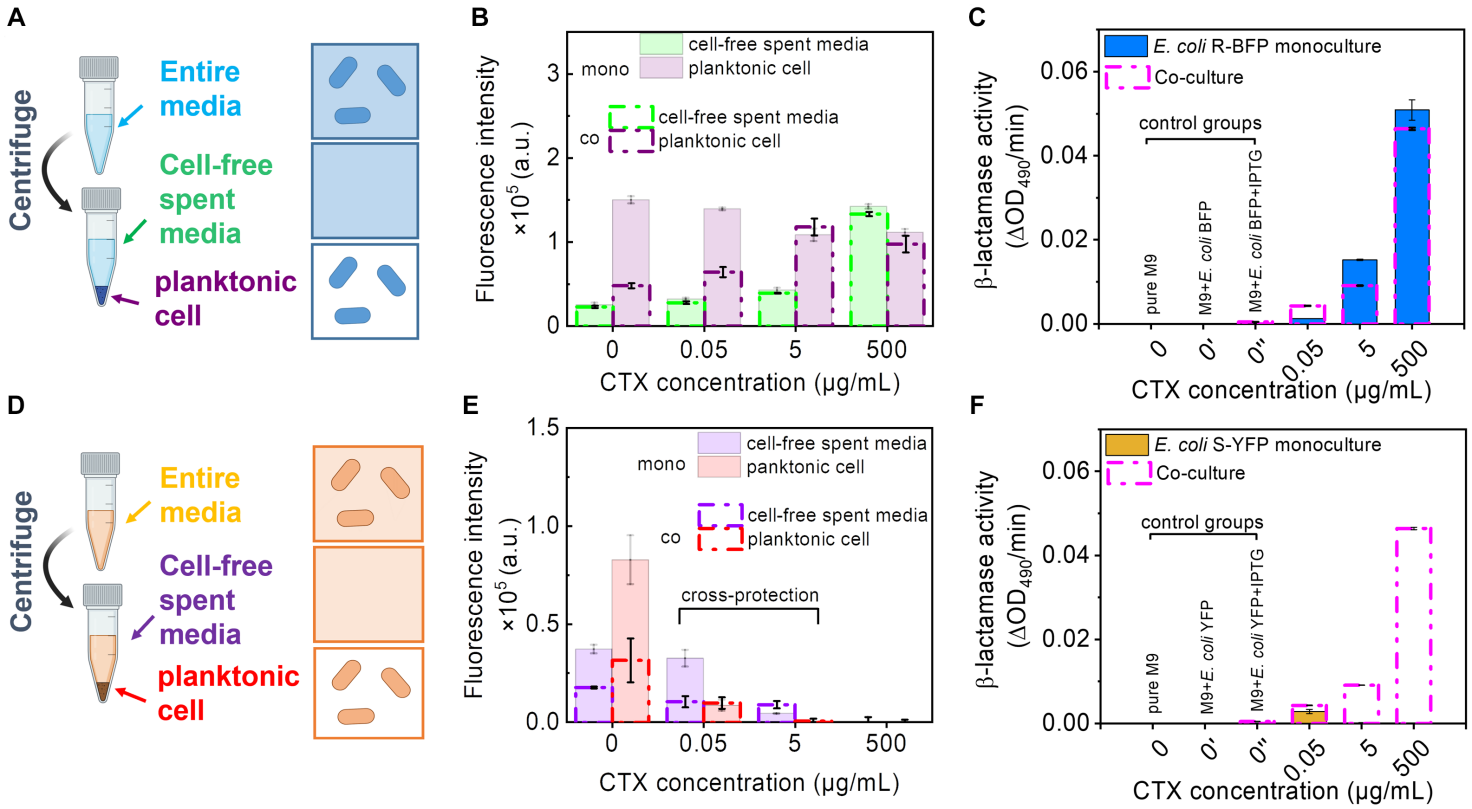
Fluorescence intensity and β-lactamase activity detection of monoculture and co-culture of *E. coli* R-BFP and *E. coli* S-YFP. **(A)** Separating the *E. coli* R-BFP entire bacterial medium (incubating for 20 h) to cell-free media and pure cells by centrifuging, filtering, and individually collecting. **(B)** Comparison of fluorescence intensity of *E. coli* BFP (*Em* = 400, *Ex* = 460 nm) from cell-free spent media and planktonic cells of monoculture and co-culture (with *E. coli* S-YFP with initial cell biomass ratio 1:1) with CTX concentrations of 0, 0.05, 5, and 500 μg/mL. **(C)** β-lactamase activity testing of *E. coli* R-BFP monoculture and co-culture (with *E. coli* S-YFP with initial cell biomass ratio 1:1) cell-free spent media in 96 well plates with various concentrations of CTX (0, 0.05, 5, and 500 μg/mL,). **(D)** Separating the *E. coli* S-YFP entire cell medium (incubating for 20 h) to cell-free media and planktonic cells by centrifuging, filtering, and individually collecting. **(E)** Comparison of fluorescence intensity of *E. coli* YFP (*Em* = 505, *Ex* = 535 nm) from cell-free spent media and planktonic cells of monoculture and co-culture (with *E. coli* R-BFP with initial cell biomass ratio 1:1) with CTX concentrations of 0, 0.05, 5, and 500 μg/mL. **(F)** β-lactamase activity testing of *E. coli* S-YFP monoculture and co-culture (with *E. coli* R-BFP with initial inoculum cell biomass ratio 1:1) cell-free spent media in 96 well plates with various concentrations of CTX (0, 0.05, 5, and 500 μg/mL). Red absorbance (OD_490_) varies with the hydrolysis of nitrocefin by β-lactamase, so the β-lactamase activity was determined by measuring the change in red absorption per minute (measured for 1 h) in the plate reader. All error bars are standard deviations.

The previous results (Figure 3) suggest that filamentous cells may be more permeable and prone to lysis (Wong et al., 2021), resulting in the leakage of fluorescent protein and enzymes into the environment, which would enhance cross-protection. To investigate whether filaments exhibit greater permeability than planktonic cells, we measured the fluorescence intensity of the entire media, cell-free spent media, and only planktonic cells. After 20 h of incubation, the entire culture media was collected (with a fraction reserved for measuring the overall culture performance) and then separated into cells (dispensed in fresh M9 media) and cell-free spent media (Figures 4A,D). Subsequently, we measured the fluorescence of the entire media, cells, and the cell-free spent media. In low CTX concentrations (from 0 to 0.05 μg/mL), *E. coli* R-BFP fluorescence in co-culture decreased to half the value of monoculture (Figure 4B), as before (Figure 2). However, when antibiotic concentration exceeded the MIC of *E. coli* S-YFP (0.0625 μg/mL), the fluorescence of *E. coli* R-BFP from cell and cell-free media remained at similar levels (Figure 4B) in both co-culture and monoculture, suggesting R-BFP is the only strain surviving in co-culture.

With a further increase in CTX concentrations from 5 to 500 μg/mL, *E. coli* R-BFP survived, but the fluorescent signal gradually shifted from cells (purple bars) to cell-free medium (green bars, Supplementary Figures S14C,E). This shift suggests that the high antibiotic concentration damages the cell walls, leading to lysis and possibly enhanced permeability of surviving filaments and planktonic cells, which subsequently causes the release of fluorescent protein into the media.

In addition to fluorescence (Figure 4B, green lines), we also assessed the β-lactamase activity of the cell-free spent media. The β-lactamase activity of the cell-free spent media exhibited a similar trend to that of fluorescence (Figure 4C, blue bar). In the absence of CTX, cells seem to maintain their normal size and shape (*ca.* 2 μm in length and 0.2 μm in diameter) with relatively low permeability compared to cells in higher CTX concentrations. Consequently, the β-lactamase activity detected in the 0 cefotaxime + IPTG treatment is not apparent. Specifically, the release of β-lactamase from *E. coli* R-BFP reached its peak around 25 μg/mL, followed by a gradual decline at higher CTX levels (Supplementary Figure S15B) due to cell death.

To further investigate the influence of cell morphology (filamentation) on extracellular β-lactamase activity at high CTX levels, we examined β-lactamase release in relation to filamentous cell frequency (Supplementary Figure S2). At low CTX concentrations (0–0.5 μg/mL), β-lactamase exhibited relatively low levels in both cell-free filtrate and in planktonic cells (intra-cells), as shown in Supplementary Figure S2E. As the CTX concentration increased (5 μg/mL to 500 μg/mL), β-lactamase activity measured from both cell-free filtrate (released) and entire culture media (represent total production, after the cell wall break treatment, including released β-lactamase activity from cell-free filtrate and β-lactamase activity intra planktonic cells) concomitantly increased (almost 10 times). The fraction of β-lactamase released (cell-free filtrate/entire cell media) changed with rising CTX levels and closely mirrored the pattern of cell filamentation frequency in response to antibiotic concentration (Supplementary Figure S2F). These findings suggest that the high β-lactamase activity production might be correlated to the high CTX concentrations, and the increased β-lactamase activity release fraction may be caused by the high incidence of filamentous cells at high CTX concentrations. This highlights that the changes in cell morphology play a role in the enhanced extracellular β-lactamase activity (releasing in cell-free media and degrading CTX throughout the culture to protect S-YFP in co-culture), possibly due to: (i) increased β-lactamase leakage caused by greater cell wall permeability (Georgiou et al., 1988), (ii) release from lysed filaments, or (iii) a combination of both mechanisms.

### 3.5 Disparity in β-lactamase activity influences cross-protection window

**Figure 5:**
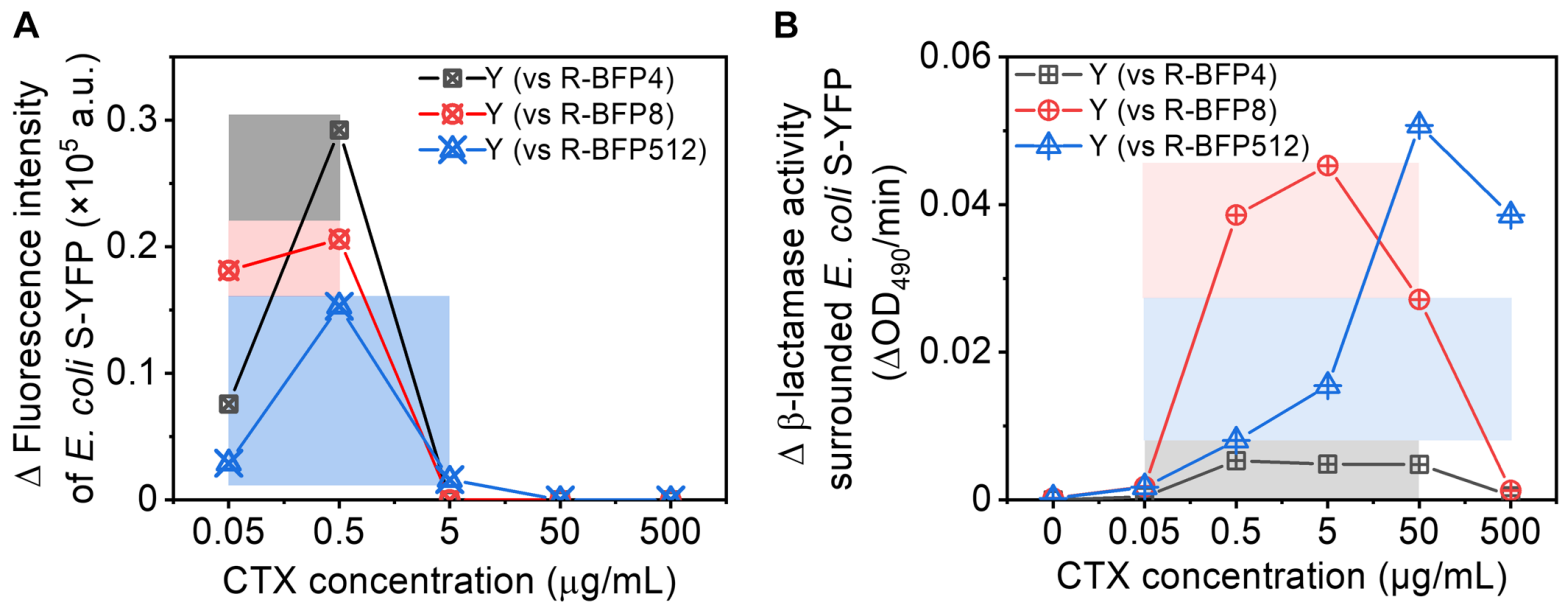
Comparison of cross-protection windows between *E. coli* S-YFP and various *E. coli* R-BFP strains (with varying MICs): **(A)** Net increase in fluorescence (a bulk signal from the population) in *E. coli* S-YFP planktonic cells (difference between co-culture and monoculture); The cross-protection windows represented by colored rectangles (darker colors) refer to the survival of S-YFP (increased growing cells) due to antibiotic degradation by R-BFP in the coexisting environment. **(B)** Net increase in β-lactamase activity (difference from cell-free spent media between co-culture and monoculture). The cross-protection window represented by the colored rectangles (lighter colors) indicates that R-BFP degrades the antibiotic (increased β-lactamase activity) and may contribute to S-YFP survival.

Cross-protection typically occurs within a limited range of CTX concentrations, which is closely tied to the β-lactamase activity - and hence MIC - of the resistant strains. In this work, we have observed cross-protection between two bacterial strains, one (*E. coli* R-BFP512) expressing a β-lactamase with high catalytic hydrolysis efficiency of CTX, the other (*E. coli* S-YFP) not, resulting in a *ca.* 9,000-fold different MIC. To investigate the influence of the disparity in β-lactamase activity on cross-protection, we examined co-cultures comparing sensitive *E. coli* S-YFP and three *E. coli* R-BFP strains with MICs of 4 μg/mL (64-fold MIC difference relative to S-YFP, R-BFP4), 8 μg/mL (128-fold MIC difference, R-BFP8), and 512 μg/mL (9,000-fold MIC difference, R-BFP512), respectively. By comparing the growth of *E. coli* S-YFP cells (Figure 5A) and β-lactamase activity (Figure 5B) in both co-culture and monoculture conditions, we observed that smaller β-lactamase activity differences resulted in more effective cross-protection at lower antibiotic concentrations (0.05 to 0.5 μg/mL), manifested by more fluorescent planktonic cells appearance (Figure 5A). Higher β-lactamase activity allowed protection to emerge at higher antibiotic concentrations, thereby shifting the cross-protection window toward higher concentrations (0.05 to 5 μg/mL). While higher enzyme activity positively influences bacterial survival, at high antibiotic concentrations (up to 50 ug/mL), sensitive bacteria may not be able to survive before the enzyme degrades antibiotics in the culture medium, because high concentrations of antibiotics can damage the cell wall within minutes (see Supplementary Figure S16; Geyrhofer et al., 2023).

Therefore, sensitive strains can survive in an environment with antibiotic concentrations above their MIC levels through cross-protection, which depends on the β-lactamase activity differences (different catalytic activities of β-lactamase) and antibiotic dosages (sensitive bacteria survive the attack before β-lactamase deactivate antibiotics).

## 4 Conclusion

In this work, we addressed the conditions of cross-protection of antibiotic-sensitive bacteria by bacteria expressing clinically relevant antibiotic-degrading β-lactamase enzymes. We found that the cross-protection window for the antibiotic-sensitive *E. coli* S-YFP in the presence of a resistant strain expressing a highly active β-lactamase extended to antibiotic concentrations *ca.* 100 times higher than the MIC of the sensitive strain. Further analyzes revealed that filamentation of bacterial cells triggered by antibiotic stress might contribute to the observed cross-protection by releasing their β-lactamases through cell lysis and/or leakage facilitated by more permeable cell walls. Enzyme activity analysis showed that the increased β-lactamase activity of the resistant strain in the co-culture environment deactivated CTX was responsible for cross-protecting the antibiotic-sensitive strain. Notably, we demonstrate that the cross-protection window depends on both the antibiotic dosage and the difference in β-lactamase activity between co-cultured strains. Whether bacterial filaments enhance the evolution and spread of antibiotic resistance through enhanced survival of bacterial populations (Bos et al., 2015), or rather frustrate resistance evolution by enhancing competition from susceptible bacteria, is an important question for future research.

Our observations were made using high throughput nanoliter droplet reactors, which enabled simultaneous monitoring of both coexisting bacteria in a large number of replicate cultures (up to 500). The application of nanoliter droplet bioreactors enables the quantitative characterization of high-resolution population-dynamic processes, such as during bacterial competition and cooperation. Insight is pivotal for advancing our comprehension of the evolution and transmission of antibiotic resistance, ultimately contributing to more effective strategies for managing this pressing global concern.

## Data availability statement

The original contributions presented in the study are included in the article/Supplementary material, further inquiries can be directed to the corresponding author.

## Author contributions

XZ: Formal analysis, Investigation, Methodology, Writing – original draft. PR: Investigation, Methodology, Writing – review & editing. AF: Methodology, Writing – review & editing. JV: Conceptualization, Writing – review & editing. LB: Conceptualization, Funding acquisition, Investigation, Writing – review & editing.

## References

[ref1] Adamowicz E. M. Flynn J. Hunter R. C. Harcombe W. R. (2018). Cross-feeding modulates antibiotic tolerance in bacterial communities. ISME J. 12, 2723–2735. doi: 10.1038/s41396-018-0212-z, PMID: 29991761 PMC6194032

[ref2] Aminov R. I. (2010). A brief history of the antibiotic era: lessons learned and challenges for the future. Front. Microbiol. 1:134. doi: 10.3389/fmicb.2010.00134, PMID: 21687759 PMC3109405

[ref3] Andrews J. M. (2001). Determination of minimum inhibitory concentrations. J. Antimicrob. Chemother 48, 5–16. doi: 10.1093/jac/48.suppl_1.5 11420333

[ref4] Angelis D. Giulia P. D. G. Posteraro B. Sanguinetti M. Tumbarello M. (2020). Molecular mechanisms, epidemiology, and clinical importance of β-lactam resistance in Enterobacteriaceae. Int. J. Mol. Sci. 21:5090. doi: 10.3390/ijms21145090, PMID: 32708513 PMC7404273

[ref5] Antimicrobial Resistance Collaborators (2022). Global burden of bacterial antimicrobial resistance in 2019: a systematic analysis. Lancet (London, England) 399, 629–655. doi: 10.1016/S0140-6736(21)02724-0, PMID: 35065702 PMC8841637

[ref6] Balaban N. Q. Merrin J. Chait R. Kowalik L. Leibler S. (2004). Bacterial persistence as a phenotypic switch. Science 305, 1622–1625. doi: 10.1126/science.1099390, PMID: 15308767

[ref7] Baraban L. Bertholle F. Salverda M. L. M. Bremond N. Panizza P. Baudry J.. (2011). Millifluidic droplet analyser for microbiology. Lab Chip 11, 4057–4062. doi: 10.1039/c1lc20545e, PMID: 22012599

[ref8] Bell M. (2014). Antibiotic misuse: a global crisis. JAMA Intern. Med. 174, 1920–1921. doi: 10.1001/jamainternmed.2014.3289, PMID: 25285726

[ref9] Bos J. Zhang Q. Vyawahare S. Rogers E. Rosenberg S. M. Austin R. H. (2015). Emergence of antibiotic resistance from multinucleated bacterial filaments. Proc. Natl. Acad. Sci. U. S. A. 112, 178–183. doi: 10.1073/pnas.1420702111, PMID: 25492931 PMC4291622

[ref10] Bottery M. J. Pitchford J. W. Friman V.-P. (2021). Ecology and evolution of antimicrobial resistance in bacterial communities. ISME J. 15, 939–948. doi: 10.1038/s41396-020-00832-7, PMID: 33219299 PMC8115348

[ref11] Diene S. M. Pinault L. Keshri V. Armstrong N. Khelaifia S. Chabrière E.. (2019). Human Metallo-β-Lactamase Enzymes Degrade Penicillin. Sci. Rep. 9:12173. doi: 10.1038/s41598-019-48723-y 31434986 PMC6704141

[ref12] Farr A. D. Pesce D. Das S. G. Zwart M. P. De Visser J. A. G. M. (2023). The fitness of Beta-lactamase mutants depends nonlinearly on resistance level at sublethal antibiotic concentrations. MBio 14:e00098. doi: 10.1128/mbio.00098-23, PMID: 37129484 PMC10294655

[ref13] Filipiak M. Łoś J. M. Łoś M. (2020). Efficiency of induction of Shiga-toxin lambdoid prophages in *Escherichia coli* due to oxidative and antibiotic stress depends on the combination of prophage and the bacterial strain. J. Appl. Genet. 61, 131–140. doi: 10.1007/s13353-019-00525-8, PMID: 31808108 PMC6968986

[ref14] Frost I. Smith W. P. J. Mitri S. Millan A. S. Davit Y. Osborne J. M.. (2018). Cooperation, competition and antibiotic resistance in bacterial colonies. ISME J. 12, 1582–1593. doi: 10.1038/s41396-018-0090-4, PMID: 29563570 PMC5955900

[ref15] GBD 2019 Diseases and Injuries Collaborators (2020). Global burden of 369 diseases and injuries in 204 countries and territories, 1990-2019: a systematic analysis for the global burden of disease study 2019. Lancet (London, England) 396, 1204–1222. doi: 10.1016/S0140-6736(20)30925-9 33069326 PMC7567026

[ref16] Georgiou G. Shuler M. L. Wilson D. B. (1988). Release of periplasmic enzymes and other physiological effects of Beta-lactamase overproduction in *Escherichia coli*. Biotechnol. Bioeng. 32, 741–748. doi: 10.1002/bit.260320603, PMID: 18587779

[ref17] Geyrhofer L. Ruelens P. Farr A. D. Diego Pesce J. de Visser A. G. M. Brenner N. (2023). Minimal surviving inoculum in collective antibiotic resistance. MBio 14:e02456. doi: 10.1128/mbio.02456-22 37022160 PMC10128016

[ref18] Geyrhofer L. Ruelens P. Farr A. D. Pesce D. De Visser J. A. G. M. Brenner A. N. (2022). Race to survival during antibiotic breakdown determines the minimal surviving population size. bioRxiv 2022:2802. doi: 10.1101/2022.08.04.502802

[ref19] Gomes L. Monteiro G. Mergulhão F. (2020). The impact of IPTG induction on plasmid stability and heterologous protein expression by *Escherichia coli* biofilms. Int. J. Mol. Sci. 21:E576. doi: 10.3390/ijms21020576, PMID: 31963160 PMC7013871

[ref20] Gullberg E. Albrecht L. M. Karlsson C. Sandegren L. Andersson D. I. (2014). Selection of a multidrug resistance plasmid by sublethal levels of antibiotics and heavy metals. MBio 5:14. doi: 10.1128/mBio.01918-14, PMID: 25293762 PMC4196238

[ref21] Justice S. S. Hunstad D. A. Cegelski L. Hultgren S. J. (2008). Morphological plasticity as a bacterial survival strategy. Nat. Rev. Microbiol. 6, 162–168. doi: 10.1038/nrmicro1820, PMID: 18157153

[ref22] Kim S. W. Park S. B. Im S. P. Lee J. S. Jung J. W. Gong T. W.. (2018). Outer membrane vesicles from β-lactam-resistant *Escherichia coli* enable the survival of β-lactam-Susceptible *E. coli* in the presence of β-lactam antibiotics. Sci. Rep. 8:5402. doi: 10.1038/s41598-018-23656-0 29599474 PMC5876404

[ref23] Lima L. M. Silva B. N. M. Barbosa G. Barreiro E. J. (2020). β-Lactam antibiotics: an overview from a medicinal chemistry perspective. Eur. J. Med. Chem. 208:112829. doi: 10.1016/j.ejmech.2020.112829 33002736

[ref24] Maddocks J. L. May J. R. (1969). Indirect pathogenicity of penicillinase-producing enterobacteria in chronic bronchial infections. Lancet 293, 793–795. doi: 10.1016/S0140-6736(69)92063-7, PMID: 4180358

[ref25] Medaney F. Dimitriu T. Ellis R. J. Raymond B. (2016). Live to cheat another day: bacterial dormancy facilitates the social exploitation of β-lactamases. ISME J. 10, 778–787. doi: 10.1038/ismej.2015.154, PMID: 26505830 PMC4817691

[ref26] Munita J. M. Arias C. A. (2016). Mechanisms of antibiotic resistance. Microbiology Spectrum 4:15. doi: 10.1128/microbiolspec.VMBF-0016-2015, PMID: 27227291 PMC4888801

[ref27] Nguyen-Le T. A. Zhao X. Bachmann M. Philip Ruelens J. De Visser A. G. M. Baraban L. (2023). High-throughput gel microbeads as incubators for bacterial competition study. Micromachines 14:645. doi: 10.3390/mi14030645 36985052 PMC10058504

[ref28] Nicoloff H. Andersson D. I. (2016). Indirect resistance to several classes of antibiotics in cocultures with resistant bacteria expressing antibiotic-modifying or -degrading enzymes. J. Antimicrob. Chemother. 71, 100–110. doi: 10.1093/jac/dkv312 26467993

[ref29] Palzkill T. (2018). Structural and mechanistic basis for extended-Spectrum drug-resistance mutations in altering the specificity of TEM, CTX-M, and KPC β-lactamases. Front. Mol. Biosci. 5:16. doi: 10.3389/fmolb.2018.00016 29527530 PMC5829062

[ref30] Pande S. Merker H. Bohl K. Reichelt M. Schuster S. de Figueiredo L. F.. (2014). Fitness and stability of obligate cross-feeding interactions that emerge upon gene loss in bacteria. ISME J. 8, 953–962. doi: 10.1038/ismej.2013.211, PMID: 24285359 PMC3996690

[ref31] Ruelens P. De Visser J. A. G. M. (2021). Choice of β-lactam resistance pathway depends critically on initial antibiotic concentration. Antimicrob. Agents Chemother. 65, e00471–e00421. doi: 10.1128/AAC.00471-21 33972257 PMC8284463

[ref32] Ruiz J. (2018). Etymologia: TEM. Emerg. Infect. Dis. 24:709. doi: 10.3201/eid2404.ET2404

[ref33] Saebelfeld M. Das S. G. Hagenbeek A. Krug J. De Visser J. A. G. M. (2022). Stochastic establishment of β-lactam-resistant *Escherichia coli* mutants reveals conditions for collective resistance. Proc. R. Soc. B Biol. Sci. 289:20212486. doi: 10.1098/rspb.2021.2486 PMC9065960 35506221

[ref34] Schenk M. F. Szendro I. G. Salverda M. L. M. Krug J. De Visser J. A. G. M. (2013). Patterns of epistasis between beneficial mutations in an antibiotic resistance gene. Mol. Biol. Evol. 30, 1779–1787. doi: 10.1093/molbev/mst096, PMID: 23676768 PMC3708503

[ref35] Vega N. M. Gore J. (2014). Collective antibiotic resistance: mechanisms and implications. Curr. Opin. Microbiol. Antimicrob. 21, 28–34. doi: 10.1016/j.mib.2014.09.003 PMC4367450 25271119

[ref36] Windels E. M. Meriem Z. B. Zahir T. Verstrepen K. J. Hersen P. Van den Bergh B.. (2019). Enrichment of Persisters enabled by a SS-lactam-induced filamentation method reveals their stochastic single-cell awakening. Commun. Biol. 2, 1–7. doi: 10.1038/s42003-019-0672-3 31815194 PMC6884588

[ref37] Wong F. Wilson S. Helbig R. Hegde S. Aftenieva O. Zheng H.. (2021). Understanding Beta-lactam-induced lysis at the single-cell level. Front. Microbiol. 12:7. doi: 10.3389/fmicb.2021.712007, PMID: 34421870 PMC8372035

[ref38] Yoon J. H. Shin J.-H. Park J. H. Park T. H. (2015). Effect of light intensity on the correlation between cell mass concentration and optical density in high density culture of a filamentous microorganism. Korean J. Chem. Eng. 32, 1842–1846. doi: 10.1007/s11814-015-0012-3

[ref39] Yu J. S. L. Correia-Melo C. Zorrilla F. Herrera-Dominguez L. Wu M. Y. Hartl J.. (2022). Microbial communities form rich extracellular metabolomes that Foster metabolic interactions and promote drug tolerance. Nat. Microbiol. 7, 542–555. doi: 10.1038/s41564-022-01072-5, PMID: 35314781 PMC8975748

[ref40] Yurtsev E. A. Conwill A. Gore J. (2016). Oscillatory dynamics in a bacterial cross-protection mutualism. Proc. Natl. Acad. Sci. U. S. A. 113, 6236–6241. doi: 10.1073/pnas.1523317113, PMID: 27194723 PMC4896713

[ref41] Zahir T. Wilmaerts D. Franke S. Weytjens B. Camacho R. Marchal K.. (2020). Image-based dynamic phenotyping reveals genetic determinants of filamentation-mediated β-lactam tolerance. Front. Microbiol. 11:374. doi: 10.3389/fmicb.2020.00374 32231648 PMC7082316

[ref42] Zhao X. Illing R. Ruelens P. Bachmann M. Gianaurelio C. De Visser J. A.. (2021). Coexistence of fluorescent *Escherichia coli* strains in Millifluidic droplet reactors. Lab Chip 21, 1492–1502. doi: 10.1039/D0LC01204A, PMID: 33881032

