# Supplementary material for: Population dynamics of cross-protection against β-lactam antibiotics in droplet microreactors
Source: Front Microbiol. 2023 Dec 21;14:1294790. doi: 10.3389/fmicb.2023.1294790 (PMC10773670; doi:10.3389/fmicb.2023.1294790)
Supplement: Supplementary file 1 [file Data_Sheet_1.docx]

Supplementary Material.

# Supplementary Figures and Tables

##
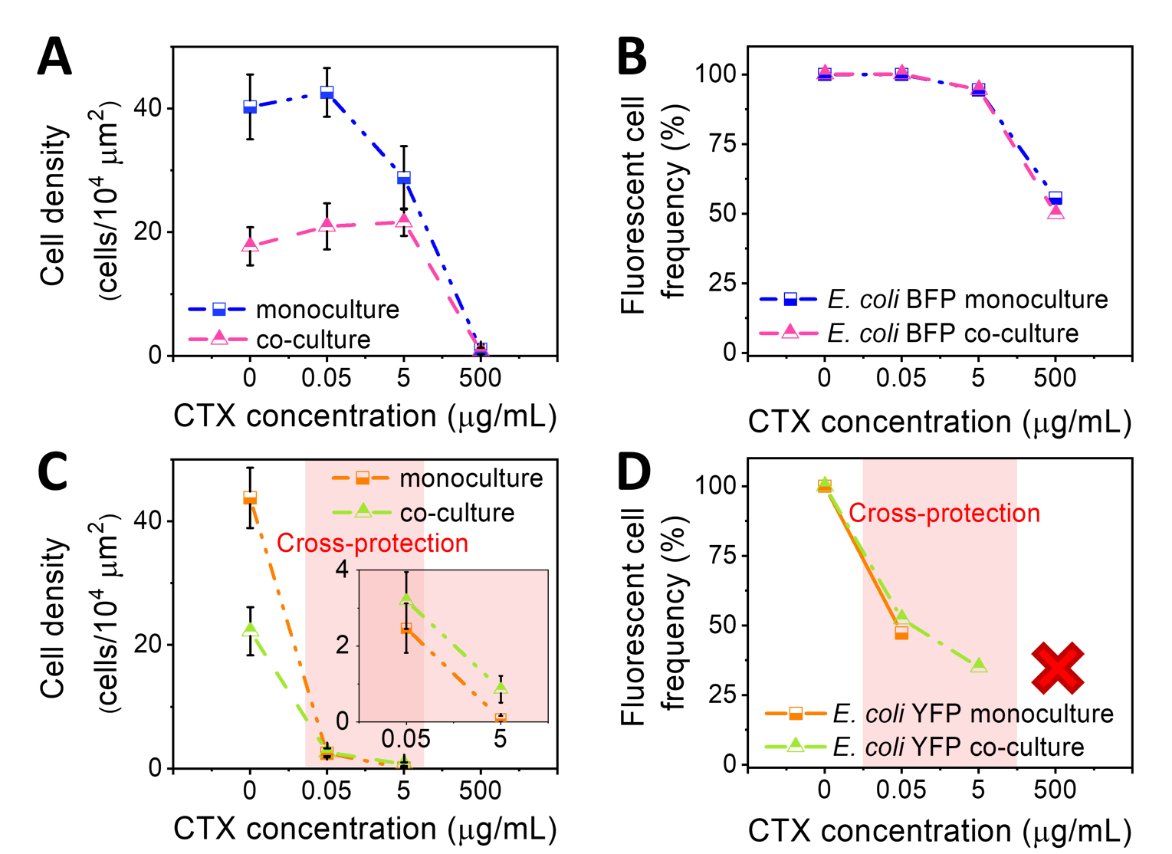
Supplementary Figures

**Supplementary Figure 1.** **Cell number and status analysis: counting cell number and checking cell fluorescence under a microscope after incubation for 20 hours.** *E. coli* R-BFP monoculture and co-culture (with *E. coli* S-YFP with initial cell biomass ratio 1:1): **(A)** cell density calculated by counting cell numbers per area on a glass slide (represented as in the area of 10000 µm^2^), and **(B)** fluorescence cell frequency of monoculture and co-culture *E. coli* R-BFP. *E. coli* S-YFP monoculture and co-culture (with *E. coli* R-BFP with initial cell biomass ratio 1:1): **(C)** cell density, and **(D)** fluorescence cell frequency (Fluorescent cells as a percentage of total cells) of monoculture and co-culture *E. coli* S-YFP. The total cell number (n) used to analyze cell status in monoculture and co-culture for each CTX condition is shown in **Supplementary Table 1**. Error bars are standard deviation from 8 replicates.


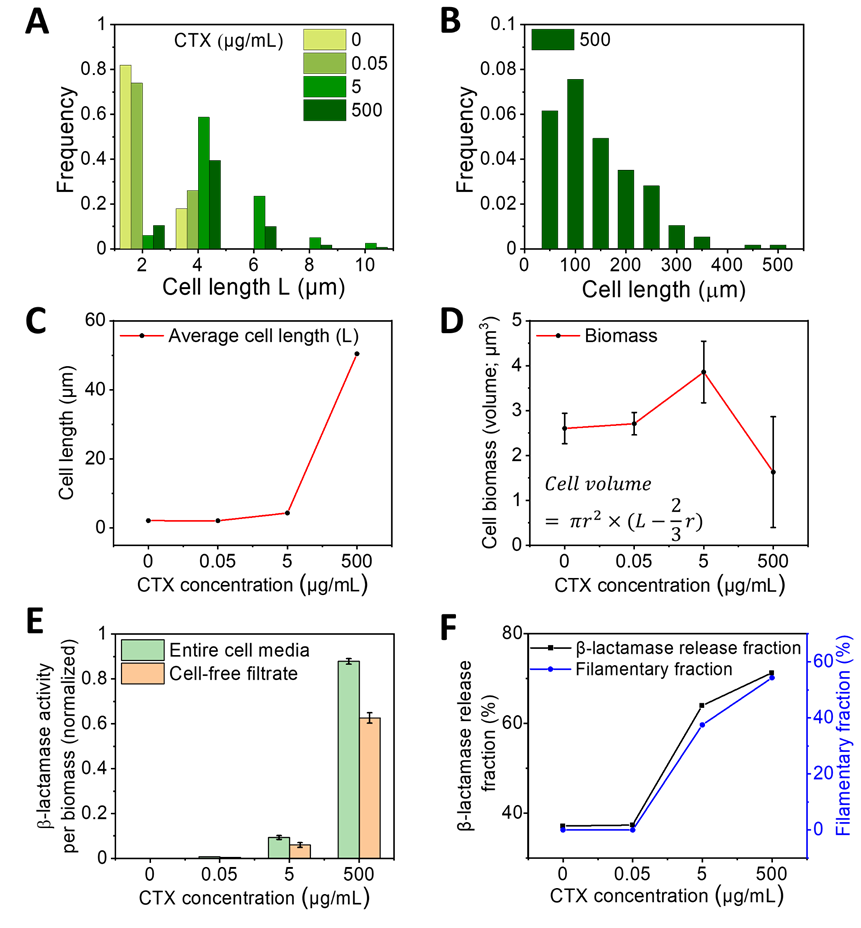


**Supplementary Figure 2.** Comparison of the **(A, B)** cell length distribution, **(C)** average cell length, and **(D)** cell biomass of *E. coli* R-BFP512 monoculture with different concentrations of CTX (initial cell biomass equal to 1000 cells/droplet). The cell biomass was calculated based on the cell diameter (D = 0.2 µm = 2⋅r), cell length (L), and cell density (**Fig.3-figure supplement 11A**). Cell biomass = cell volume × cell density. Error bars are standard deviation from 3 replicates. **(E)** β-lactamase activity per biomass (normalized) of production (entire cell media after cell wall break treatment) and release (cell-free spent media, filtrate), for each measurement has 8 replicates (n=8). **(F)** Comparison of β-lactamase release fraction (cell-free filtrate/entire cell media) and filamentary fraction in varying CTX concentrations.


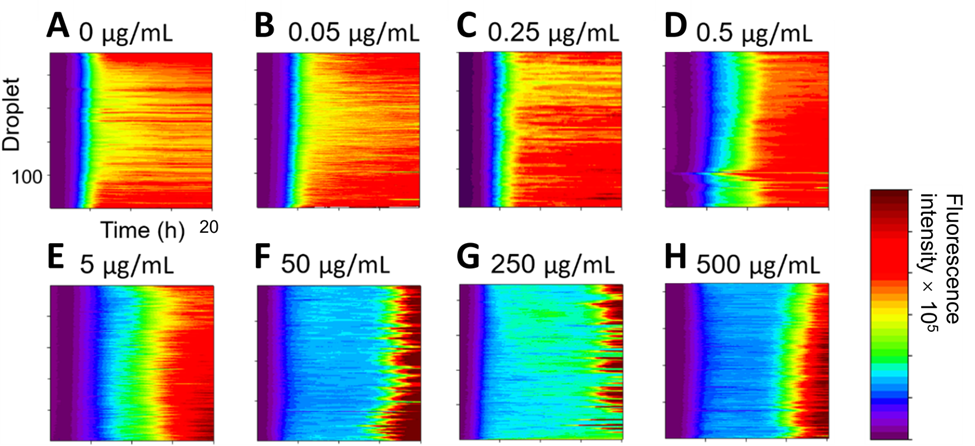


**Supplementary Figure 3.** **Fluorescence heat map of *E. coli* R-BFP monoculture with different CTX concentrations**: (**A)** 0 µg/mL, **(B)** 0.05 µg/mL, **(C)** 0.25 µg/mL, **(D)** 0.5 µg/mL, **(E)** 5 µg/mL, **(F)** 50 µg/mL, (**G)** 250 µg/mL and **(H)** 500 µg/mL. All groups were monoculture with an initial cell density of 1000 cells/droplet. The fluorescence intensity scale is from 0.0-3.0 × 10^5^ a.u., as shown at the bottom right.


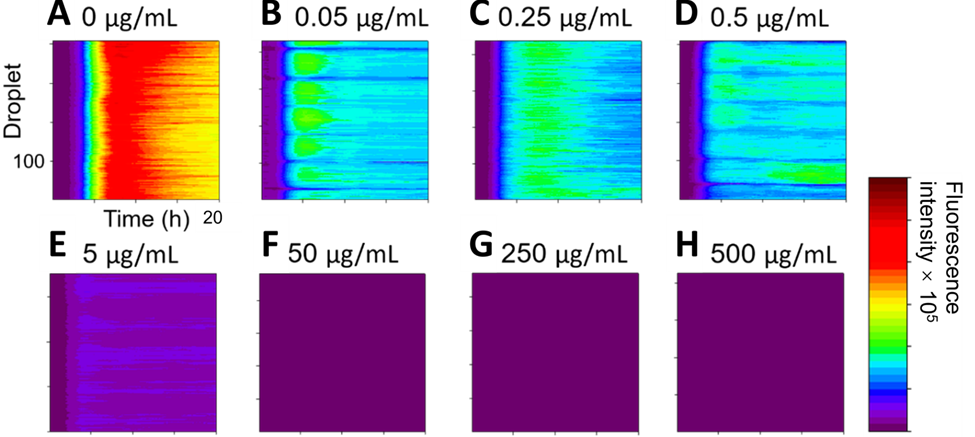


**Supplementary Figure 4.** **Fluorescence heat map of *E. coli* S-YFP monoculture with different CTX concentration**s: **(A)** 0 µg/mL, **(B)** 0.05 µg/mL, **(C)** 0.25 µg/mL, **(D)** 0.5 µg/mL, **(E)** 5 µg/mL, **(F)** 50 µg/mL, **(G)** 250 µg/mL and **(H)** 500 µg/mL. All groups were monoculture with an initial cell density of 1000 cells/droplet. The fluorescence intensity scale is from 0.0-3.0 × 10^5^ a.u., as shown at the bottom right.

**
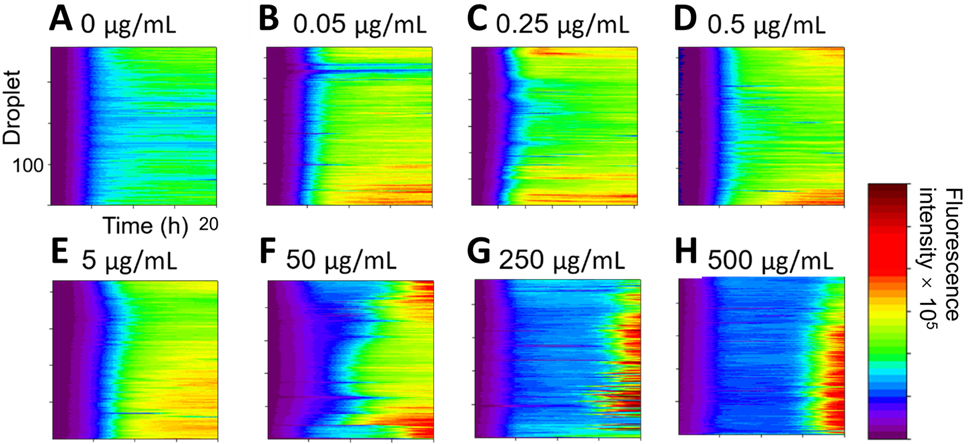
**

**Supplementary Figure 5.** **Fluorescence heat map of *E. coli* R-BFP co-culture (with *E. coli* S-YFP of the initial cell density ratio 1:1) with different CTX concentrations**: **(A)** 0 µg/mL, **(B)** 0.05 µg/mL, **(C)** 0.25 µg/mL, **(D)** 0.5 µg/mL, **(E)** 5 µg/mL, **(F)** 50 µg/mL, **(G)** 250 µg/mL and **(H)** 500 µg/mL. The time scale is from 0 to 1200 min. The fluorescence intensity scale is from 0.0-3.0 × 10^5^ a.u., as shown at the bottom right.


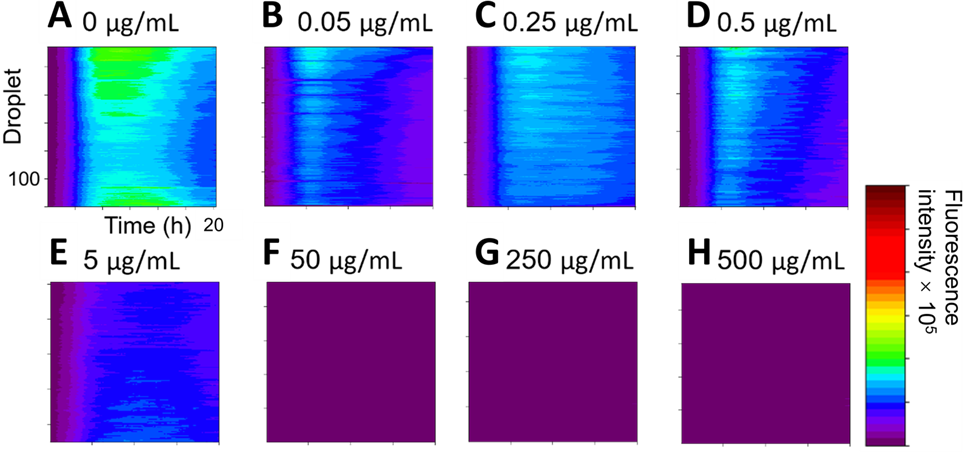


**Supplementary Figure 6.** Fluorescence heat map of *E. coli* S-YFP co-culture (with *E. coli* R-BFP of the initial cell density ratio 1:1) with different CTX concentrations: **(A)** 0 µg/mL, **(B)** 0.05 µg/mL, **(C)** 0.25 µg/mL, **(D)** 0.5 µg/mL, **(E)** 5 µg/mL, **(F)** 50 µg/mL, **(G)** 250 µg/mL and **(H)** 500 µg/mL. The time scale is from 0 to 1200 min. The fluorescence intensity scale is from 0.0-3.0 × 10^5^ a.u., as shown at the bottom right.

**
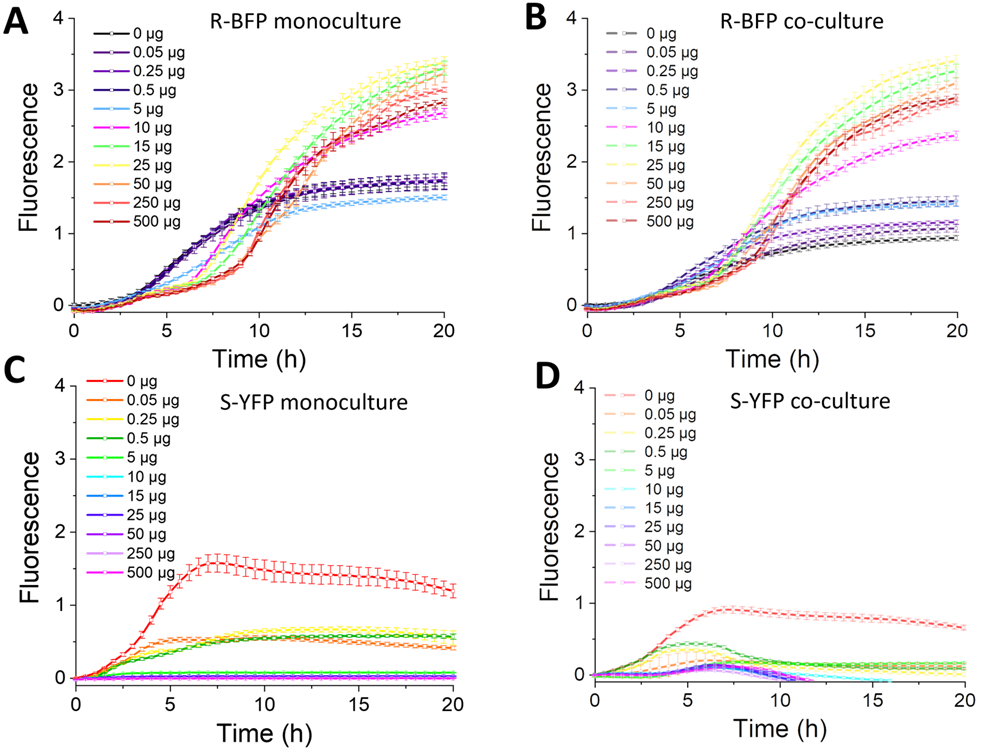
**

**Supplementary Figure 7.** Fluorescence intensity change with incubation time with various CTX concentrations of **(A)** *E. coli* R-BFP monoculture; **(B)** *E. coli* R-BFP co-culture with *E. coli* S-YFP; **(C)** *E. coli* S-YFP monoculture; **(D)** *E. coli* S-YFP co-culture with *E. coli* R-BFP. All monoculture groups were prepared with an initial cell density of 1000 cells/droplet in the droplet (200 nL). All co-culture groups were prepared with an initial cell biomass ratio of 1000:1000 cells/droplet. The time scale is from 0 to 20 hours. Error bars are standard deviation from 8 replicates.


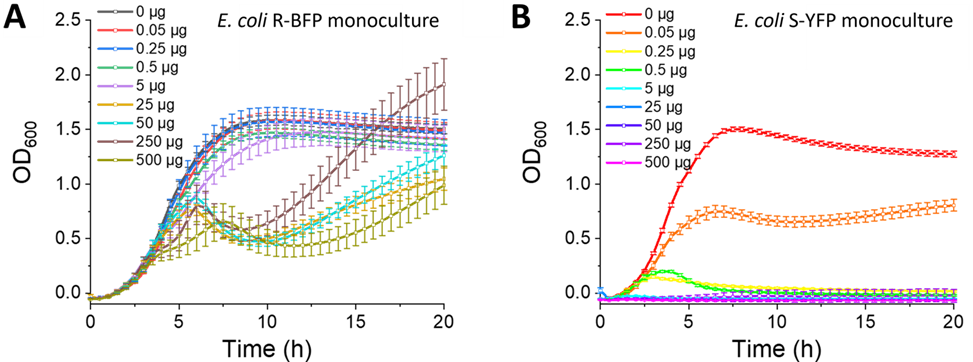


**Supplementary Figure 8.** Growth curves of monoculture *E. coli* with different concentrations of CTX measured by a plate reader (OD_600_): **(A)** *E. coli* R-BFP and **(B)** *E. coli* S-YFP.


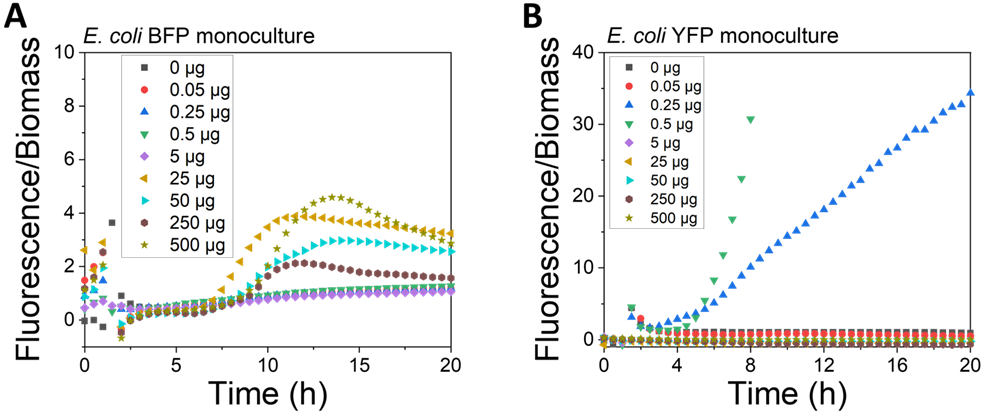


**Supplementary Figure 9.** Fluorescence intensity per biomass (OD_600_) of monoculture *E. coli* with different concentrations of CTX: **(A)** *E. coli* R-BFP and **(B)** *E. coli* S-YFP.


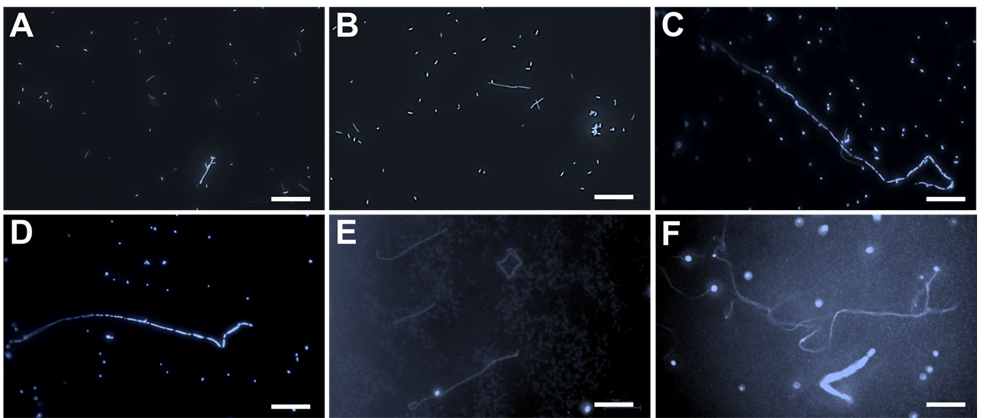


**Supplementary Figure 10.** **Monoculture of *E. coli* R-BFP morphology after incubated with different concentrations of CTX for 20 h**: (**A)** 10, **(B)** 15, **(C)** 25, **(D)** 50, **(E)** 250, and **(F)** 500 µg/mL observed with fluorescence microscopy (20 hours incubation) with 100 × magnification under blue light. All scale bars are 20 µm.


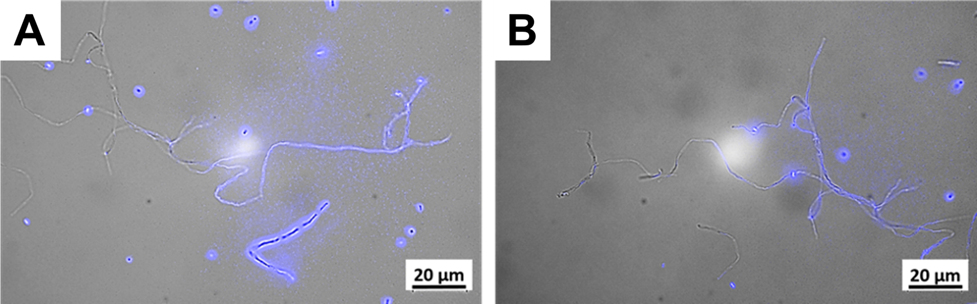


**Supplementary Figure 11.** ***E. coli* R-BFP morphology after incubated in CTX with concentration of 500 µg/mL for 20 h (observed with fluorescence microscopy):** **(A)** monoculture and **(B)** co-culture with *E. coli* S-YFP. Pictures are taken with 100× magnification and merged by fluorescence and bright field graphs. The scale bar is 20 µm.


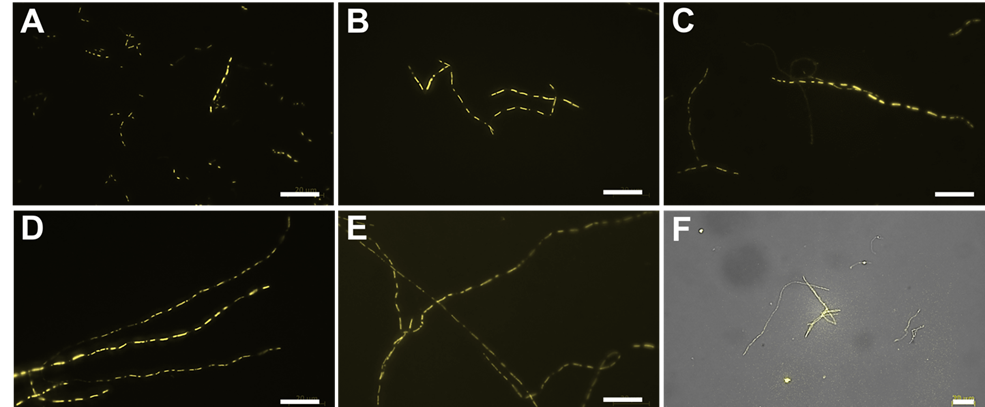


**Supplementary Figure 12.** **Monoculture of *E. coli* S-YFP morphology after incubated in CTX with different concentrations for 20 h**: **(A)** 0.005, (**B)** 0.01, **(C)** 0.015, **(D)** 0.02, **(E)** 0.05, and **(F)** 0.5 µg/mL observed with fluorescence microscopy (20 hours incubation) with 100 × magnification under blue light*.* All scale bars are 20 µm.


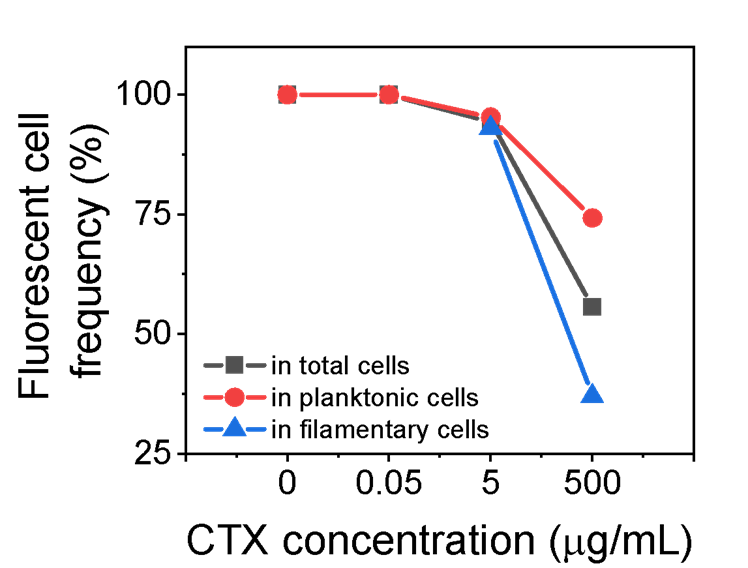


**Supplementary Figure 13.** Fluorescent cell frequency among planktonic, filamentary, and total cells of monoculture *E. coli* R-BFP with different concentrations of CTX (observed with fluorescence microscopy after 20 hours incubation with 100 × magnification under UV light). Planktonic and filamentous cells were discerned based on the cell length (cells with a cell length greater than 4 µm were considered filamentous cells).


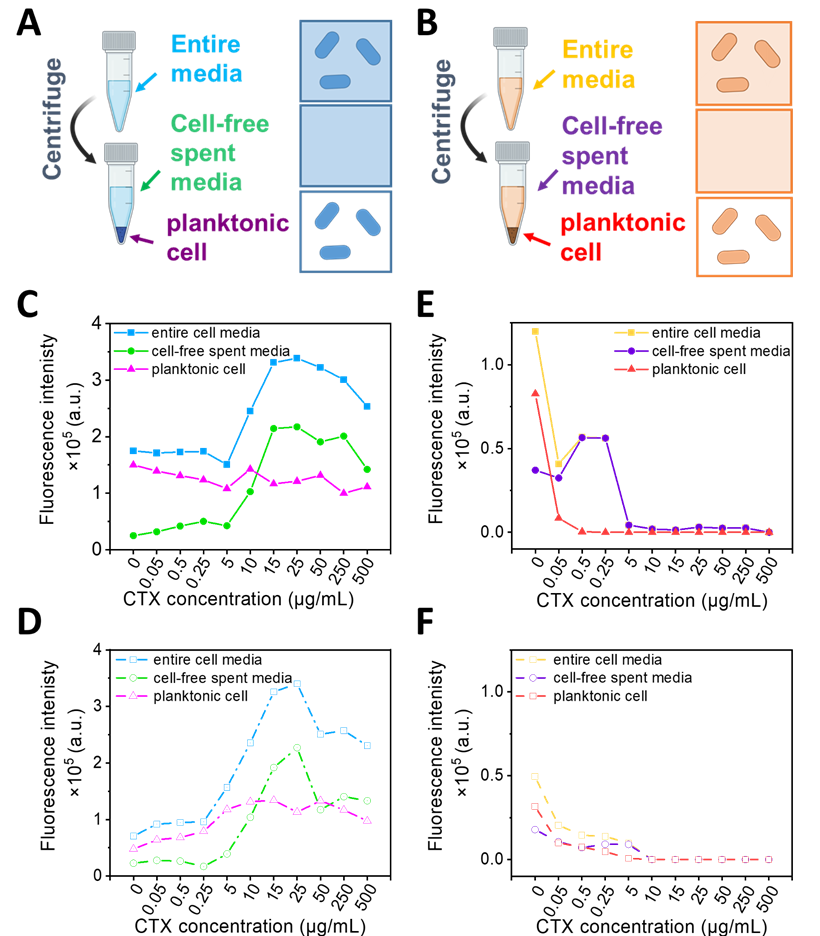


**Supplementary Figure 14.** **Fluorescence detection of entire cell media, cell-free spent media, and resuspended planktonic cell** of **(A)** and **(B)** the preparation steps, **(C)** *E. coli* R-BFP monoculture, **(D)** *E. coli* S-YFP monoculture, **(E)** *E. coli* R-BFP co-culture, and **(F)** *E. coli* S-YFP co-culture.


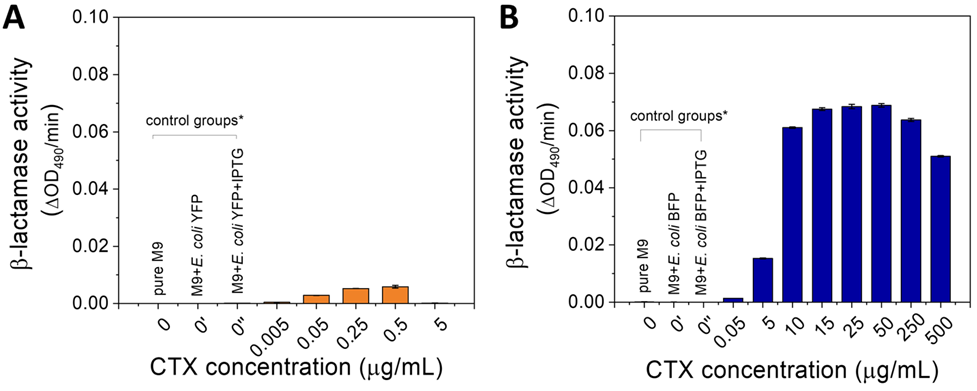


**Supplementary Figure 15.** **β-lactamase activity** of **(A)** monoculture *E. coli* S-YFP (with initial cell biomass equal 1000 cells/droplet) and **(B)** monoculture *E. coli* R-BFP (with initial cell biomass equal 1000 cells/droplet) cell-free media in 96 well plates with various concentrations of CTX. Red absorbance (OD_490_) varies with the hydrolysis of nitrocefin by β-lactamase, so the β-lactamase activity was determined by measuring the change in red absorption per minute (measured for 1 hour) in the plate reader.


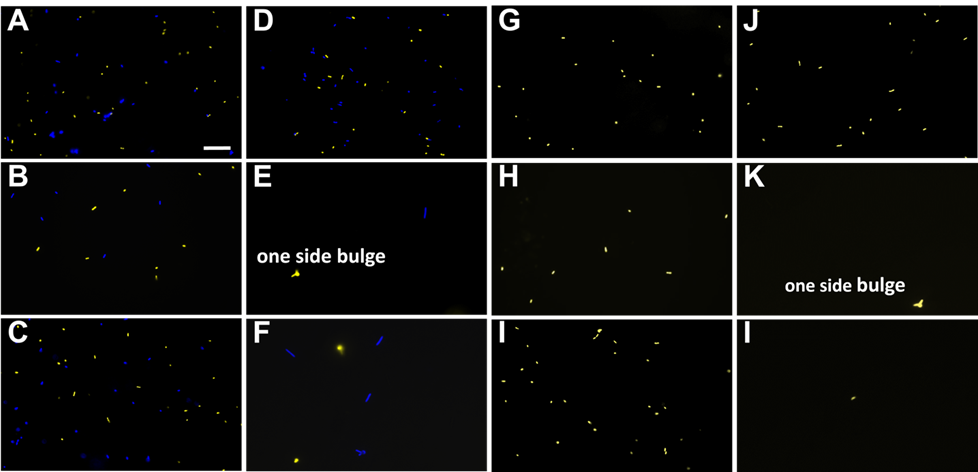


**Supplementary Figure 16.** **Monoculture and co-culture of *E. coli* R-BFP and *E. coli* S-YFP in different concentrations of CTX were observed with fluorescence microscopy (0 hours incubation) with 100× magnification.** *E. coli* R-BFP and *E. coli* S-YFP co-culture in CTX with concentrations of **(A)** 0, **(B)** 0.005, **(C)** 0.5, **(D)** 5, **(E)** 50, and **(F)** 500 µg/mL. *E. coli* S-YFP monoculture in CTX with concentrations of **(G)** 0, 0.005, **(H)** 0.5, **(I)** 5, **(J)** 50, and **(K)** 500 µg/mL. *E. coli* S-YFP cells show one side bulge once incubated in CTX with concentrations up to 50 µg/mL. The scale bar is 20 µm.

## Supplementary Tables

**Supplementary Table 1:** The total cell number (n) used to analyze cell status in monoculture and co-culture for each CTX condition after incubation for 20 h.

| Total cell number (n) | Monoculture | | Co-culture | |
| --- | --- | --- | --- | --- |
| CTX (µg/mL) | R-BFP | S-YFP | R-BFP | S-YFP |
| 0 | 1748 | 1954 | 998 | 1247 |
| 0.05 | 2017 | 258 | 1062 | 135 |
| 5 | 1819 | 32 | 1515 | 50 |
| 500 | 568 | - | 477 | - |

**Supplementary Table 2:** The total cell number (n) used to analyze cell status of *E. coli* R-BFP monoculture for each CTX condition after incubation for 20 h.

| Total cell number (n) | Filamentary cell | | | Normal cell | | | Total |
| --- | --- | --- | --- | --- | --- | --- | --- |
| CTX (µg/mL) | Fluo. | no Fluo. | total | Fluo. | no Fluo. | total |  |
| 0 | 0 | 0 | 0 | 1748 | 0 | 1748 | 1748 |
| 0.05 | 0 | 0 | 0 | 2017 | 0 | 2017 | 2017 |
| 5 | 1094 | 83 | 1177 | 612 | 30 | 642 | 1819 |
| 500 | 118 | 200 | 318 | 186 | 64 | 250 | 586 |
